# GABA, Glx, and GSH in the cerebellum: their role in motor performance and learning across age groups

**DOI:** 10.3389/fnagi.2025.1626417

**Published:** 2025-07-03

**Authors:** Shanti Van Malderen, Melina Hehl, Stefanie Verstraelen, Robbe Breugelmans, Georg Oeltzschner, Stephan P. Swinnen, Koen Cuypers

**Affiliations:** ^1^Neuroplasticity and Movement Control Research Group, Rehabilitation Research Institute (REVAL), Hasselt University, Diepenbeek, Belgium; ^2^KU Leuven, Leuven Brain Institute (LBI), Leuven, Belgium; ^3^Movement Control and Neuroplasticity Research Group, Department of Movement Sciences, Group Biomedical Sciences, KU Leuven, Heverlee, Belgium; ^4^Translational MRI, Department of Imaging and Pathology, KU Leuven, Leuven, Belgium; ^5^Imo-imomec, Hasselt University, Hasselt, Belgium; ^6^Medicine, Department of Radiology and Radiological Science, Johns Hopkins, Baltimore, MD, United States

**Keywords:** magnetic resonance spectroscopy, cerebellum, aging, gamma-aminobutyric acid, Glx, glutathione

## Abstract

**Introduction:**

The cerebellum is essential for motor control and learning, relying on structural and functional integrity. Age-related atrophy leads to Purkinje cell loss, but subtle neurochemical changes in GABA, Glx (glutamate + glutamine), and glutathione (GSH) may precede degeneration and contribute to motor decline.

**Methods:**

25 younger (YA) and 25 older adults (OA) were included in this study. Magnetic resonance spectroscopy (MRS), using the MEGA-PRESS sequence, was used to investigate how age affects GABA, Glx and GSH levels in the right cerebellar hemisphere, and their relationship with motor performance, measured using a visuomotor bimanual tracking task (BTT).

**Results:**

In line with previous work YA outperformed OA on both the simple and complex task variants of the BTT. Furthermore, YA demonstrated faster short-term motor learning as compared to OA. On the metabolic level, no significant age group differences in cerebellar GABA, Glx or GSH levels, nor any task-related modulation of GABA or Glx were observed. Additionally, neither baseline neurometabolite levels nor their modulation predicted motor performance or learning.

**Discussion:**

These results align with previous research suggesting that neurometabolic aging is region-specific, with the cerebellum potentially being more resilient due to its slower aging process. Since neither baseline nor task-related modulation of GABA, Glx, or GSH predicted motor performance or learning, cerebellar neurometabolite concentrations may not directly underlie age-related behavioral changes. Instead, volumetric decline and changes in structural and functional connectivity in the aging cerebellum may play a more significant role in motor decline as compared to neurochemical alterations. Nonetheless, it is important to consider that motor performance and learning rely on distributed brain networks—including cortical and subcortical structures—which also undergo age-related changes and may contribute to observed behavioral declines. While our findings do not support a direct role of cerebellar neurometabolite levels in age-related motor performance differences, they underscore the complexity of neurochemical aging.

## 1 Introduction

The cerebellum plays a vital role in a wide range of functions, including motor control, particularly in tasks involving coordination, such as bimanual movements and visuomotor tracking (Miall et al., 2001; Debaere et al., 2004; e.g., Boisgontier et al., 2018) as well as motor learning (e.g., D'Angelo, 2018; Hull and Regehr, 2022). Similar to cortical brain regions, the cerebellum undergoes atrophic changes with advancing age (Raz et al., 2010; Ramanoel et al., 2018; Han et al., 2020). In particular, cerebellar gray matter (GM) (Andersen et al., 2003; Yu et al., 2017) and white matter (WM) (Raz et al., 2005) have been reported to decrease across the entire cerebellum with advancing age. Nevertheless, GM loss exhibits a spatiotemporal heterogeneous pattern in which right lobule V and bilateral lobule VIIIa—distinct areas of motor representation (Buckner et al., 2011)—showed higher rates of GM loss as compared to the overall rate (Yu et al., 2017). Age-related volumetric changes in the cerebellum manifest in significant declines in motor coordination and motor learning (Raz et al., 2000; e.g., Boisgontier et al., 2018). Specifically, Boisgontier et al. (2018) showed that cerebellar GM loss directly correlates with bimanual coordination performance deficits in older adults, highlighting the key role of cerebellar degeneration in broader behavioral impairments.

The decline in cerebellar volume coincides with significant neuronal loss, as the cerebellum undergoes a 40% loss of both Purkinje and granule cells (Andersen et al., 2003), generally beginning between the ages of 50 and 60 years (Hall et al., 1975). In addition to the apotheosis of Purkinje cells, research in cats and rodents also shows degeneration of Purkinje cells with increasing age (Woodruff-Pak et al., 2010; Zhang et al., 2010; Childs et al., 2021). This loss and degradation of Purkinje cells has been suggested to relate to behavioral changes in animal models like Lurcher mutant mice—an animal model of cerebellar degeneration—, which show impaired motor skills, reduced motor learning, and deficits in spatial orientation and associative learning (Hilber and Caston, 2001; Porras-García et al., 2005; Cendelín et al., 2008; Porras-García et al., 2010). However, not all dysfunctions can be attributed to volumetric changes or Purkinje cell loss and degradation alone. Subtler structural or biochemical changes often precede neuronal death, as seen in Staggerer mutant mice, where motor and working memory impairments emerge even when Purkinje cell numbers remain intact (Caston et al., 2003; Caston et al., 2004). This suggests that, alongside other factors such as neural functional changes (Seidler et al., 2010; Bernard and Seidler, 2014; Bernard et al., 2021), early alterations in cerebellar biochemical properties may significantly contribute to the progressive decline in motor and cognitive functions with age. Despite evidence of structural cerebellar decline with age, recent studies using cerebellar brain inhibition (CBI) have demonstrated functional resilience of cerebellar excitability in older adults (Mooney et al., 2022; Van Malderen et al., 2025). Furthermore, the use of the same bimanual tracking task in Van Malderen et al. (2025) as in the present study supports its suitability for engaging the cerebellum in older adults (Boisgontier et al., 2018). Yet, age-related differences in cerebellar neurochemical processes and their relationship with motor control and motor learning in humans remain largely unexplored.

As Purkinje cells, which are the main output neurons of the cerebellar hemispheres, use gamma-aminobutyric acid (GABA)ergic neurotransmission to exert inhibitory control via their projections (Sastry et al., 1997), optimal GABAergic function can be considered to play a vital role in cerebellar function (Gao et al., 2006). GABA is the primary inhibitory neurotransmitter in the human brain, and its importance in human motor behavior and motor learning has already been demonstrated in cerebral regions (e.g., Boy et al., 2010; Maes et al., 2022; for a review see: Li et al., 2022).

As glutamate (Glu) is the principal excitatory neurotransmitter in the human brain, its role is equally vital for proper cerebellar function. In the cerebellum, granule cells, mossy fibers, and part of the neurons of the deep cerebellar nuclei–constituting the main class of excitatory projections to the thalamus–primarily utilize glutamate as their neurotransmitter (Bentivoglio and Kuypers, 1982; Bentivoglio and Molinari, 1986; Otis et al., 1997; Ruigrok et al., 2015). Optimal glutamatergic signaling is thus essential for the modulation and coordination of motor output. The importance of cerebellar glutamate in motor performance is highlighted, among other things, by the disruption of the cerebellar error correction mechanism observed in neurodegenerative cerebellar disorders affecting motor control, such as essential tremor (Tapper et al., 2020; also see their Figure 4). Due to the difficulty in spectrally separating glutamate from glutamine in magnetic resonance spectroscopy (MRS) at 3 T, their concentrations are often reported as a combined measure referred to as Glx (Bell et al., 2021).

**Figure 1:**
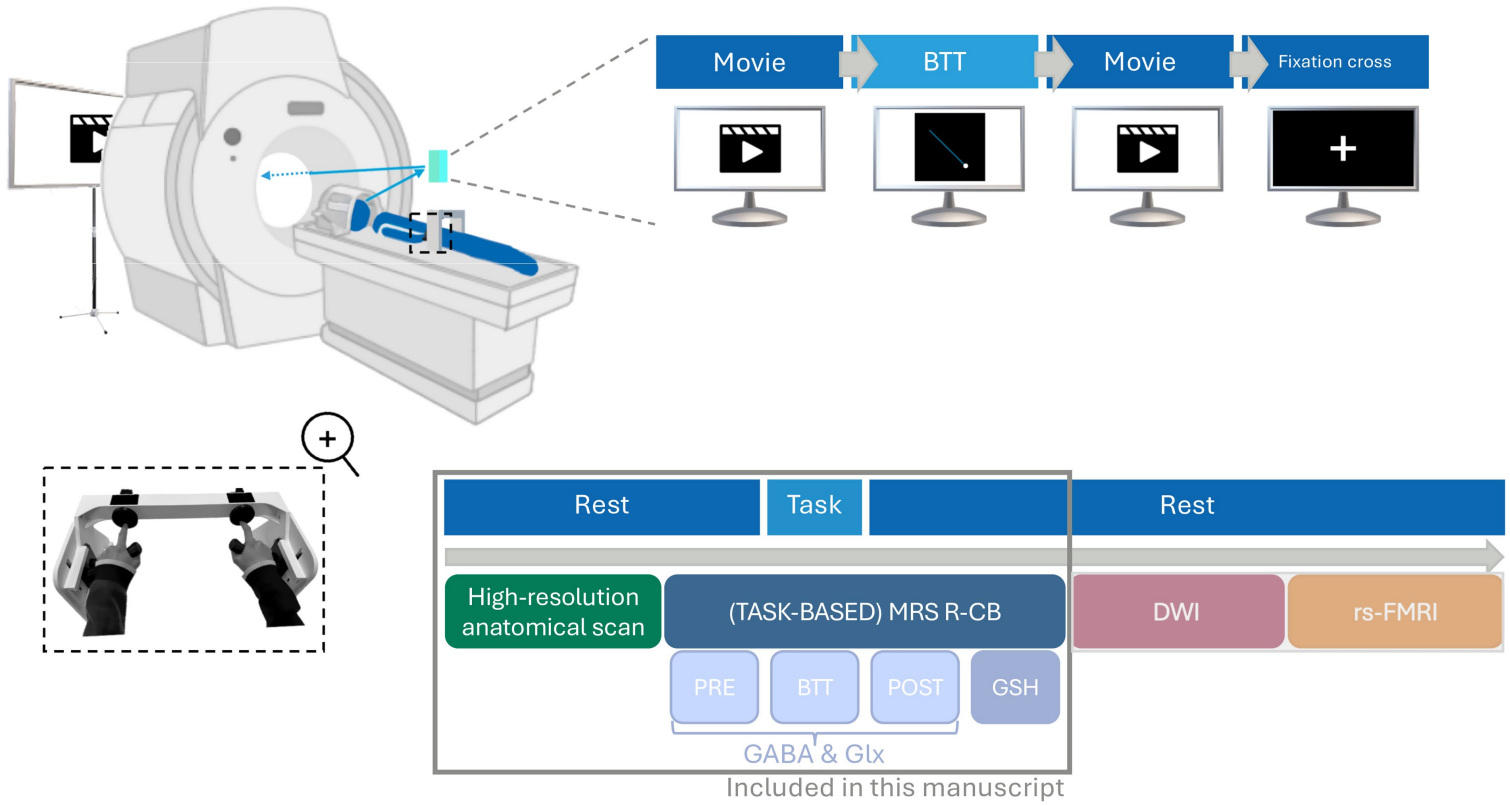
Overview of the magnetic resonance imaging (MRI) session (105 min). Participants participated in a 105-min MRI session, which included a short break dividing the session into two parts. The first part involved acquiring high-resolution T1- and T2-weighted anatomical images (~10 min) followed by magnetic resonance spectroscopy (MRS) measurements (~4 × 11 min) of the right cerebellar hemisphere (R-CB). The second part included diffusion-weighted imaging (DWI; ~15 min) and resting-state functional MRI (rs-fMRI; ~15 min). During the acquisition of anatomical scans and the first MRS measurement assessing resting gamma-aminobutyric acid (GABA_pre_) and glutamine and glutamate (Glx_pre_ levels) (PRE), participants watched a movie. This was followed by a task-based MRS acquisition (BTT), where participants performed a bimanual tracking task (BTT) while GABA and Glx levels were measured. The final two MRS measurements, assessing GABA_post_, Glx_post_ and glutathione (GSH) were conducted at rest again (POST and GSH), allowing participants to resume watching the movie. During the second part, they remained at rest while watching a movie during the DWI acquisition, followed by looking at a white cross on a black background during the rs-fMRI acquisition. This manuscript focuses solely on MRS measurements and the anatomical T1-weighted scans necessary for overlay and segmentation, as indicated by the gray box in the figure. BTT, bimanual tracking task; DWI, diffusion-weighted imaging; GABA, gamma-aminobutyric acid; Glx, combined measure for glutamine and glutamate; GSH, glutathione; MRI, magnetic resonance imaging; MRS, magnetic resonance spectroscopy; R-CB, right cerebellar hemisphere; Rs-fMRI, resting -state functional magnetic resonance imaging.

In addition to baseline (resting state) GABA and Glx levels, the modulation of GABA and Glx levels during motor execution (task-related) is suggested to play a vital role, as motor performance and skill acquisition through short-term learning are dependent on the adaptive changes in GABA and Glx within task-specific brain regions (Marsman et al., 2017; Maes et al., 2022; Li et al., 2024b; Hehl et al., 2025; Rodríguez-Nieto et al., 2025). Nevertheless, GABA and Glx levels appear to change with age (Huang et al., 2017; Porges et al., 2017a; Zuppichini et al., 2024), and these changes have been associated with the decline of various motor behaviors (Zahr et al., 2008; Porges et al., 2017a; Hermans et al., 2018a; Marenco et al., 2018; Cassady et al., 2019; Chamberlain et al., 2021; Krishnamurthy et al., 2022; Pasanta et al., 2023). However, most research has focused on cortical regions such as the primary sensorimotor cortex (SM1) and supplementary motor areas, but evidence suggests that GABA levels are differentially linked to motor performance depending on the specific brain region (e.g., Puts et al., 2011; Hermans et al., 2018a; Maes et al., 2021; Li et al., 2022). Specifically, GABA levels in a given brain region are associated with motor performance in tasks that critically depend on that particular region. While Long et al. (2015) demonstrated the feasibility and reproducibility of cerebellar GABA-edited MRS in older adults, the relationship between motor behavior and potential age-related differences in both baseline cerebellar GABA and Glx levels, as well as their task-related modulation, remains unexplored. Given the cerebellum’s vulnerability to atrophic changes and its importance in motor control and learning, it is essential to investigate whether age-related changes in GABA and Glx levels contribute to impaired motor coordination and learning.

Previous studies investigating age-related differences in cortical GABA+ and Glx levels have yielded inconsistent findings. Particularly, while some have reported lower GABA+ levels in older compared to younger adults (e.g., Chalavi et al., 2018; Cassady et al., 2019; Chamberlain et al., 2021; Maes et al., 2021; Heise et al., 2022), others found no significant group differences (e.g., Hermans et al., 2018b; Maes et al., 2018; Cuypers et al., 2021). Similarly, findings on Glx are mixed, with some reporting cortical Glx declines (e.g., Huang et al., 2017; Levin et al., 2019), while others observed no age-related differences (e.g., Weerasekera et al., 2020). These discrepancies may be due to regional variation in age differences in neurometabolite levels (Rodríguez-Nieto et al., 2023; Li et al., 2024a). However, direct evidence for age-related differences in cerebellar GABA+ and Glx concentrations remains sparse, highlighting the importance of examining these neurochemicals in the aging cerebellum.

In addition to GABA and Glx, assessing glutathione (GSH)—the brain’s primary antioxidant that protects neurons from oxidative damage—is highly valuable, particularly in the context of neurodegenerative processes such as aging (Chakrabarti et al., 2011; Mazzetti et al., 2015). This is especially relevant for the cerebellum, and particularly the granule layer, given its vulnerability to oxidative stress (Wang et al., 2009; Wang and Michaelis, 2010). Specifically, while most brain neurons demonstrate resilience to oxidative stress, neurons in specific regions, such as the hippocampal CA1 area and the cerebellar granule cell layer, are notably susceptible (Wilde et al., 1997; Wang et al., 2005; Wang et al., 2009). This heightened sensitivity to oxidative stress has been suggested to potentially contribute to the pronounced loss of cerebellar granule neurons observed in older adults (Andersen et al., 2003), potentially impacting cerebellar function over time.

While some studies report lower GSH levels in certain cortical regions with aging (e.g., Emir et al., 2011; Suri et al., 2017; Hu et al., 2024), others have found higher cortical GSH levels in the medial frontal and sensorimotor cortices of healthy older adults (Hupfeld et al., 2021). Interestingly, elevated GSH levels in the sensorimotor cortex were negatively associated with motor performance (Hupfeld et al., 2021). These mixed findings highlight inconsistencies in the current literature regarding the impact of aging on GSH levels and their functional significance remains insufficiently explored. Given that GSH levels have been found to decrease in spinocerebellar ataxia (Doss et al., 2015), it remains an open question whether similar changes occur in the aging cerebellum and, if so, how this relates to motor function. Hence, investigating GSH levels alongside GABA in the cerebellum may provide a more comprehensive understanding of the neurochemical changes associated with aging and their impact on motor control.

Both GABA and GSH can be quantified in humans using MRS, a noninvasive imaging technique that allows for the estimation of regional metabolite concentrations *in vivo*. Specifically, the MEscher–GArwood Point RESolved Spectroscopy (MEGA-PRESS) sequence (Mescher et al., 1998) enables the selective measurement of low-concentration metabolites like GABA and GSH by isolating their signals from those of more abundant overlapping metabolites.

This study aims to address key gaps in the literature by examining age-related changes in cerebellar GABA (both baseline measures and its modulation) and GSH levels and their associations with motor control and short-term motor learning. Specifically, we aim to address whether (1) GABA, Glx and GSH levels differ between younger and older adults; (2) GABA and Glx levels are being modulated during task performance and whether this task-related modulation of GABA and/or Glx differs between age groups; and whether motor performance and short-term motor learning are predicted by (3a) resting GABA, Glx or GSH levels, or (3b) task-related GABA and/or Glx modulation. By addressing these questions, this study seeks to elucidate the neurochemical basis of age-related motor control changes and the role of the cerebellum in these processes.

Based on evidence of age-related reductions in cortical GABA and Glx levels (Huang et al., 2017; Porges et al., 2017a; Maes et al., 2021; Zuppichini et al., 2024; Rodríguez-Nieto et al., 2025), alterations in their task-related modulation (Maes et al., 2022; Rodríguez-Nieto et al., 2025), and the association of these changes with impaired motor control (e.g., Hermans et al., 2018a; Cassady et al., 2019; Maes et al., 2021)—as well as findings of declining GSH levels (in some but not all studies) with age (e.g., Emir et al., 2011; Suri et al., 2017; Hu et al., 2024)—we hypothesized that:

1. Resting levels of GABA, Glx, and GSH differ between older and younger adults, consistent with prior findings in cortical motor regions.
2. GABA and Glx levels are modulated during task performance (2a), in line with previous research in cortical brain regions, with younger adults showing different task-related modulation than older adults (2b), as seen in Maes et al. (2022) for example.
3. Motor performance and short-term motor learning are predicted by resting levels of GABA, Glx, and GSH (3a), and task-related modulation of GABA and/or Glx (3b).

## 2 Methods

### 2.1 Participants

A total of 50 right-handed participants distributed across two age groups—younger adults (YA) aged 20 to 40 years (*N* = 25; mean±SD 25 ± 5 years) and older adults (OA) aged 60 to 80 years (N = 25; mean±SD 68 ± 5 years)—were included in this MRS study. All participants were right-handed [determined using the Edinburgh Handedness Inventory (EHI) (Oldfield, 1971); scores ranging from −100 to +100, where values above +50 indicated right-handedness; inclusion cut off ≥ 50]. All participants had normal or corrected-to-normal vision. Recruitment took place across Flanders, Belgium, through community and university channels, including social media, the research group’s website, advertisements for the experiment in lectures focused on learning after retirement, and posters. Additionally, older adults listed in the Movement Control & Neuroplasticity Research Groups’ database at KU Leuven were contacted via email or telephone.

Prior to participation, participants were extensively screened. Cognitive and executive functioning were evaluated using the Montreal Cognitive Assessment [MoCA; Nasreddine et al., 2005] using a cut-off score of ≤ 23/30 (Carson et al., 2018). To screen for self-reported depressive symptoms, participants completed the 21-item Beck Depression Inventory (BDI), with higher scores reflecting more severe depressive symptoms and using a cut-off score of >13 (Beck et al., 1961; Van der Does, 2002). The 90-item Symptom Checklist (SCL) was also administered to assess physical and psychological symptoms, with a cut-off score of >81 (Derogatis, 1975), ensuring participants were in good mental and physical health. Table 1 displays questionnaire scores and demographics per group. Finally, participants were excluded if they self-reported (a history of) central nervous system disorders, psychiatric conditions, use of medications that affect the central nervous system (e.g., sedatives, antidepressants), brain surgeries or injuries, health issues or treatments influencing the central nervous system, or a history of drug or alcohol abuse, or if the reported any contraindications to MRI (e.g., metal implants, claustrophobia).

**Table 1:** Participant demographics.

Characteristic	Younger adults *N* = 25 (13 F, 12 M)		Older adults *N* = 25 (13 F, 12 M)		Group comparison
	Median (Mean∇)	IQR (SD∇)	Median (Mean∇)	IQR (SD∇)	*p*-value WRST
Age (years)	25.44∇	5.03∇	67.60∇	5.35∇	
Questionnaires					
EHI LQ (%)	100.00	5.00	100.00	0.00	0.4870
MoCA (score /30)	29.00	2.00	29.00	2.00	0.4531
SCL (score /360)	11.00	19.00	13.00	25.00	0.9149
BDI (score /63)	4.00	6.50	4.00	7.50	0.5246
IPAQ (MET/h)	4674.00	4456.50	4635.00	6665.25	0.4849

Variables denoted by ∇ represent normally distributed data, for which the mean and standard deviation are reported. For non-normally distributed variables, the median and interquartile range are provided. Statistical analyses of non-normally distributed variables were conducted using the Wilcoxon Rank-sum test.

BDI, Beck’s depression inventory; EHI LQ, Edinburgh handedness inventory’s lateralization quotient; F, female; IPAQ, international physical activity questionnaire; IQR, interquartile range; M, male; MoCA, Montreal cognitive assessment; SCL, symptom checklist; SD, standard deviation; WRST, Wilcoxon Rank-sum test.

The study protocol was approved by the local ethics committee (Ethics Committee Research UZ/KU Leuven; reference S66028) and participants gave full written informed consent prior to study participation, according to the latest amendment of the Declaration of Helsinki (World Medical Association, 2013).

### 2.2 Overview experimental protocol

Prior to inclusion, each participant underwent a screening session to ensure eligibility. This session encompassed an evaluation of the previously outlined inclusion and exclusion criteria (see Section 2.1 Participants) and a practical familiarization phase. For the latter, participants entered a mock scanner, a non-magnetic replica of the MRI machine, to simulate the actual experimental environment. Within this mock setup, participants performed 15 trials of the Bimanual Tracking Task (BTT), focusing only on the simple (1:1) condition (see Section 2.3 Bimanual Tracking Task). This procedure allowed assessment of participants’ comfort in the scanner environment and verified participants’ ability to understand and perform the required task. If participants were comfortable in the scanner and agreed to continue participation, they were scheduled for a second session at the university hospital on a different day. This session included a 105-min MRI scan with a 5 min break in between. The first part involved acquiring high-resolution T1 and T2-weighted anatomical images (~10 min) and MRS measures (~4 × 11 min), while the second part included diffusion-weighted imaging (DWI; ~15 min) and resting-state functional MRI (rs-fMRI; ~15 min). This paper will focus solely on the data from the first part of the multimodal acquisition in order to maintain coherence and flow. The experimental design is illustrated in Figure 1.

**Figure 2:**
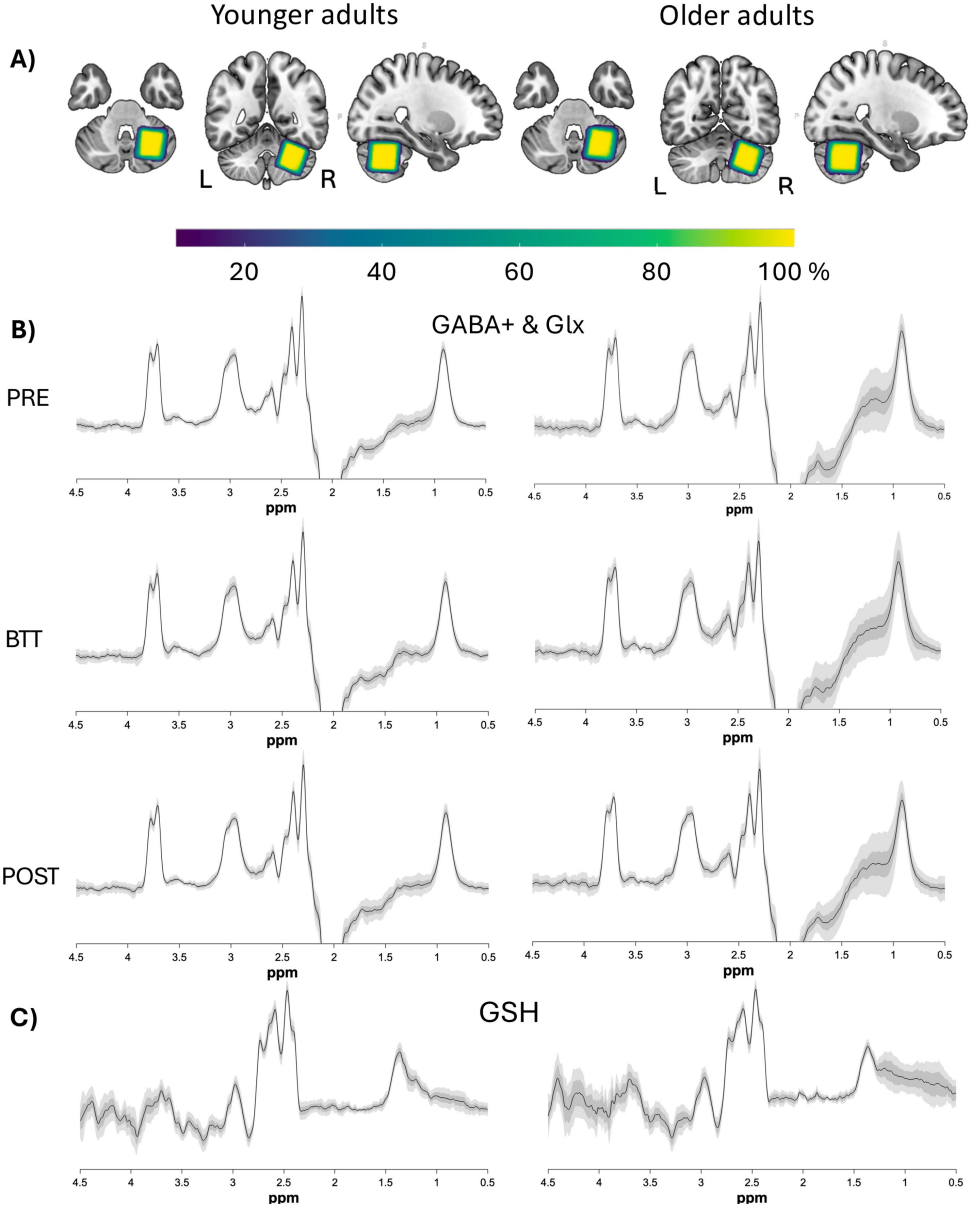
Overlay of voxels and spectra. **(A)** Normalized voxel masks projected into MNI space and overlaid on an MNI template, per age group. The colour coding indicates overlay agreement in percentage (yellow indicates a 100% overlap, whereas dark purple indicates a 10% overlap) of all voxel masks within each age group. The voxel placement was consistent, showing a high degree of overlap between participants. The images are according to the neurological convention (i.e., coronal and axial view with the right side on the right and the left side on the left of the image). **(B,C)** Difference spectra (mean±SD and 90% CI) per age group for GABA **(B)** and GSH (NAA alignment only) **(C)**. Plots for GSH are being displayed for each alignment method apart in Supplement 3. GABA, gamma-aminobutyric acid; Glx, combined measure for glutamine and glutamate; GSH, glutathione; Ppm, parts per million.

### 2.3 Bimanual tracking task (BTT)

The bimanual tracking task (BTT) was used to assess bimanual coordination and short-term motor learning since it requires real-time control of complex movement patterns with both hands. Previous studies employing a similar task design have demonstrated its sensitivity to age-related changes in bimanual coordination performance (e.g., Pauwels et al., 2015; Santos Monteiro et al., 2017; King et al., 2018; Maes et al., 2021). Originally developed by our group (Sisti et al., 2011; Pauwels et al., 2015; Monteiro et al., 2019), the set-up has since been further optimized for the MRI procedures used in this study [also see Hehl et al. (2025)]. Specifically, a non-ferromagnetic version of the BTT setup was adapted to be placed bridge-like over the participants’ hips, allowing for effortless operation while they were in a supine position.

During the task-related MRS acquisition of GABA, participants performed the BTT inside the MR scanner (± 52 trials in total over 11 min). The task was displayed via a projection screen mirrored at the participant’s eye level (projection [LCD projector: NEC NP-PA500U, 1920 × 1,200 pixels] at the cranial end of the MR scanner and viewed through a mirror sized ~14 × 9 cm, positioned approximately 13 cm from the participant’s eyes) and required participants to track a white target dot as it moved at a constant speed along a blue target line. Using coordinated movements of both index fingers, participants had to match the dot’s trajectory as accurately as possible. Each hand was placed on a handle, with the index fingers extended and positioned in the circular grooves of rotatable dials, which controlled movement along the x-axis (right finger) and y-axis (left finger). While maintaining contact with the handles, only the index fingers could move.

Each trial began with a blue target line on a black background that remained visible until the end of each trial. A large yellow dot initially covered the smaller white target dot, marking a 2000 ms anticipatory period. Participants were instructed to start moving as soon as the yellow dot disappeared, rotating the dials to control vertical (left dial) and horizontal (right dial) cursor movements, while a red line visualized the real-time trajectory of the participant’s cursor (see Supplementary material 1). The movement phase lasted 10,000 ms and ended when the white dot reached the opposite end of the blue target line. Each trial lasted 12,000 ms in total, with a 2000 ms rest between movement phases. The task comprised three conditions, each with distinct inter-hand movement frequency ratios: 1:1 (iso-frequency), 1:3, and 3:1 (non-iso-frequency). The 1:1 condition, in which both fingers moved at identical speeds, represented the least complex coordination task (e.g., Swinnen, 1998; Sisti et al., 2011). In contrast, the non-iso-frequency conditions (1:3 and 3:1) required asymmetric coordination, with, respectively, the right or left index finger moving three times faster than the other. The target moved along one of four possible trajectories: toward the upper right, upper left, lower right, or lower left quadrant, requiring different rotation directions (e.g., both hands rotating clockwise when moving to the upper right).

#### 2.3.1 BTT performance

In line with Hehl et al. (2025) and Van Malderen et al. (2025), the BTT scores (S) ranged from 0 to 100, with 100 indicating perfect performance. A preliminary score P, representing the proportion of the target line ‘covered’ by the participant’s actual movement trajectory, was first computed in accordance with Zivari Adab et al. (2020) (see their Figure 2D). More specifically, P was calculated as the total number of unique ‘completed’ points (i.e., points on the target template with a minimal Euclidean distance from the trajectory) divided by the total number of target template points, multiplied by 100. Higher scores were achieved when the participant’s red feedback line closely aligned with and paralleled the target line, resembling the correct inter-hand frequency (Zivari Adab et al., 2020). However, to penalize those who followed a parallel trajectory but deviated significantly from the target line, the preliminary score P was adjusted by a distance correction factor D, accounting for the deviation between the participant’s trajectory and the target line:


S=P⋅D

This factor, based on the average trial distance (đ) from the target line, was calculated as:


$$D=(1-\frac{đ}{8})$$

With one unit corresponding to the distance that can be covered in 200 ms.

In contrast to Hehl et al. (2025) a denominator of 8 rather than 5 was used for correction, since independent behavioral datasets suggested a denominator of 8 to be optimal for correction in a sample consisting of older and younger adults to minimize bottom and ceiling effects. D ranged from 1 (perfect alignment) to a theoretical minimum of 0 (maximal distance from the target line). However, the lower limit of D was set at 0.1 rather than 0 to allow better differentiation between participants who performed at the lower limit.

Performance was determined by averaging all scores (S) obtained throughout the 11-min block, across all conditions.

#### 2.3.2 Short-term motor learning

Short-term motor learning was quantified using the slope (a) of a linear fit (y = ax + b) to the scores (S) across trials (Hehl et al., 2025). Specifically, the linear slopes of scores (S) (dependent variable) over trials (1 – ±52; independent variable) were estimated.

### 2.4 Magnetic resonance spectroscopy (MRS) acquisition

All MRS and MRI data were acquired on a 3 Tesla scanner (Philips Achieva dstream scanner; University hospital Leuven, Gasthuisberg), with a 32-channel receiver head coil (Philips, Best, The Netherlands). The protocol consisted of an anatomical T1-weighted scan, followed by four successive MRS acquisitions of the right cerebellar hemisphere, performed during approximately 44 min, including three GABA-edited acquisitions—one at rest (GABA_pre_ and Glx_pre_), one during task performance (GABA_BTT_ and Glx_BTT_), and another at rest (GABA_post_ and Glx_post_)—and one GSH-edited acquisition (see Figure 1).

The T1-weighted high-resolution anatomical image was collected using a three-dimensional turbo field echo (3DTFE) sequence [echo time (TE) = 4.6 ms, repetition time (TR) = 9.7 ms, flip angle = 8°, field of view = 256 × 242 × 182 mm, 182 sagittal slices, voxel size = 1 × 1 × 1 mm3; acquisition time ~6 min] and was used for MRS voxel placement and correction of MRS quantification based on anatomical information.

For MRS, acquisition voxels were positioned in the right cerebellar hemisphere [30 (AP) x 25 (LR) x 25 (HF) mm^3^ = 18.75 mL], see Figure 2. Specifically, the VOI was positioned at the top of the cerebellum, aligned with the cerebellar border along the cerebrum, and placed as medially as possible to include the dentate nucleus, while avoiding contact with the fourth ventricle in both the sagittal and coronal planes. In exceptional cases—such as participant discomfort or technical issues—excessive head movement or briefly removing the participant from the scanner for an additional break required the T1-weighted anatomical scan to be repeated and the MRS voxel to be repositioned. This occurred for one older and one younger participant, where the voxel was repositioned after both the GABA_pre_ and GABA_post_ scans, and for one additional younger participant, where repositioning was necessary only after the GABA_pre_ scan.

**Figure 3:**
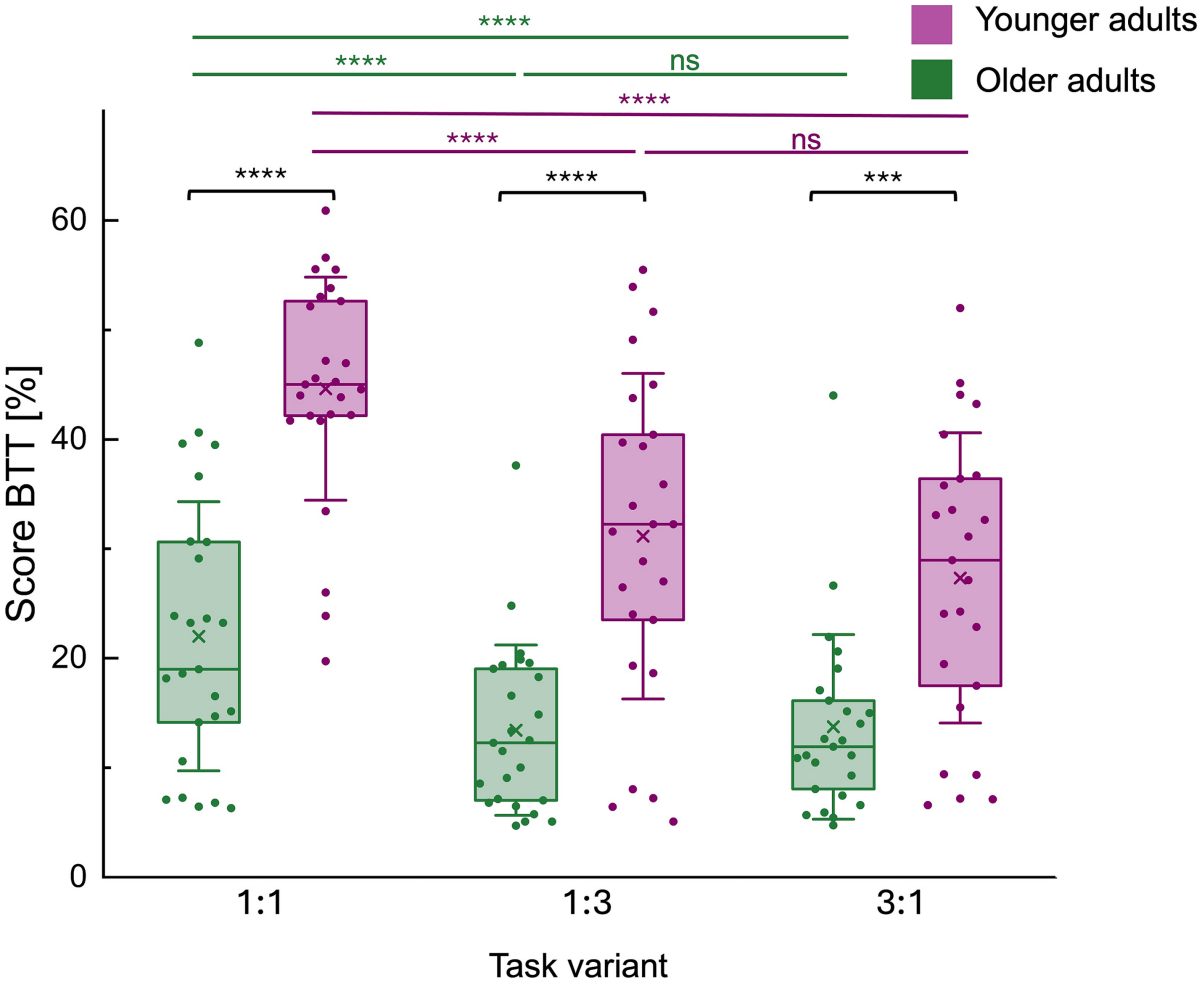
Score on the bimanual tracking task (BTT) by task variant for both age groups. Mean bimanual tracking task (BTT) performance scores per task variant (1:1, 1:3 and 3:1 frequency ratios [L: R hand]) and for each age group separately are represented by a boxplot (box = percentile 25–75, whiskers = ±1 SD, line = median, × = mean) and individual datapoints are superimposed. There was a significant main effect of age group, with younger adults (YA) outperforming older adults (OA) across all conditions. A significant main effect of condition and an age group*condition interaction were also observed. Post-hoc pairwise comparisons of the age group*condition interaction effect showed a consistent difference in performance, with younger adults outperforming older adults in all conditions. In both age groups, the 1:1 condition was consistently better executed than the 1:3 condition, and the 3:1 condition. Finally, there was no difference in the execution between the 1:3 and the 3:1 condition. **** represents *p* < 0.0001, while *** represents *p* < 0.001. BTT, bimanual tracking task; ns, non-significant.

A J-difference-edited Mescher-Garwood Point-Resolved Spectroscopy (MEGA-PRESS) protocol was used to quantify either GABA or GSH (TR = 2,000 ms, TE = 68 ms for GABA-edited / 120 ms for GSH-edited MRS, 2,000 Hz spectral width, 1,024 spectral points, ‘on’/‘off’ editing pulse frequency = 1.9/7.5 ppm for GABA-edited / 4.56/7.5 ppm for GSH-edited MRS, ‘on’/‘off’ spectra collected in an interleaved fashion, duration ~11 min per acquisition) (Mescher et al., 1998; Edden and Barker, 2007). The automated second-order “pencil-beam” (PB) shimming procedure (Philips) was used, and water suppression was applied using the Multiply Optimized Insensitive Suppression Train (MOIST; bandwidth 140 Hz). For each of the four acquisitions, an additional 16 unsuppressed water averages were acquired within the same VOI using the same acquisition parameters and later used as reference metabolite. Since macromolecular signals were co-edited during the GABA-edited MRS acquisition, the signal detected at 3 ppm represents both GABA and macromolecules, and will be referred to as GABA+.

To evaluate the accuracy and reliability of voxel placement, the overlap across participants is visualized in Figure 2. Additionally, the mean difference spectra for both age groups, along with standard deviation (SD) and confidence intervals (CI), are presented.

### 2.5 MRS data (pre)processing

A summary of all experimental procedures, in accordance with the “minimum reporting standards in MRS” consensus paper (Lin et al., 2021), is provided in Supplementary material 2.

Data processing was performed using Gannet (version 3.4.0-dev; RRID: SCR_016049) (Edden et al., 2014) and included Eddy current correction for both water and metabolite data, exponential line-broadening of 3 Hz, zero-filling to 32 K data points, and weighted averaging of the transients (Mikkelsen et al., 2020). Frequency and phase corrections were applied using robust spectral registration for all GABA+ data (Mikkelsen et al., 2020) and using spectral registration to NAA for GSH data. Since spectral registration of GSH-edited data is challenging, other alignment algorithms were used if visual inspection of pre- and post-alignment data suggested that the NAA spectral alignment algorithm was introducing error and, hence worsening spectral quality. A detailed description of this procedure can be found in Supplementary material 3.

For quantification purposes, the Gannet model for GABA+-edited spectra applies a five-parameter Gaussian-Lorentzian model to fit a Gaussian peak between 2.79 and 3.55 ppm. Specifically, the model incorporates three Gaussian peaks to represent GABA+ and the Glx [glutamate (Glu) and glutamine (Gln)] doublet between 2.79 and 4.1 ppm, along with a four-parameter curved baseline function including linear and quadratic terms. The Gannet model for GSH-edited spectra utilizes a six-parameter Gaussian model to fit the data between 2.25 and 3.5 ppm. This includes a four-parameter curved baseline function with linear and quadratic terms, one Gaussian assigned to model the GSH signal, and additional Gaussians to represent the complex co-edited aspartyl multiplet at approximately 2.6 ppm. Unsuppressed water signals were modeled using a Gaussian-Lorentzian lineshape. Consistent with previous MRS research on aging (Chalavi et al., 2018; Hermans et al., 2018b; Maes et al., 2018; Maes et al., 2021; Verstraelen et al., 2021), metabolite concentration estimates are reported relative to this internal water reference (i.e., GABA+/H2O).

MRS voxels were coregistered to the T1-weighted image using the GannetCoRegister module (SPM12), and the T1 data was then segmented with the GannetSegment module to identify the tissue fractions of gray matter (GM), white matter (WM), and cerebrospinal fluid (CSF) within the voxels (Ashburner and Friston, 2005). During quantification via the GannetQuantify module, tissue correction was applied according to the Gasparovic method (Gasparovic et al., 2006). This approach accounted for the relative MR visibility of water in different tissues (GM, WM and CSF) as well as the distinct T1 and T2 relaxation values for both water and metabolites. Modulation of GABA+ and Glx was defined as the difference between during-task (BTT) and pre-task levels, calculated by subtracting GABA_pre_ and Glx_pre_ from GABA_BTT_ and Glx_BTT_, respectively.

#### 2.5.1 Criteria for data exclusion

All data, including difference spectra before and after alignment, fit outcomes and residuals, were visually inspected. Data were excluded if they showed out-of-voxel echoes or lipid contamination. Furthermore, MRS data were excluded if no clear GABA+ or GSH signal could be detected or if modeling of the data in the Gannet toolbox failed (Edden et al., 2014). Additionally, scaled estimates for GABA+ and GSH that exceeded five times the median absolute deviation (MAD)—the average absolute distance between each data point and the median—from the median were excluded, ensuring the detection of poor fits not captured by other criteria (Rousseeuw and Hubert, 2011; Craven et al., 2022).

### 2.6 Statistical analysis

All statistical analyses were performed using SAS JMP® software (version 17, SAS Institute Inc., Cary, NC; RRID: SCR_022199) and the significance level was set at *α* = 0.05. All values are presented as mean±SD unless stated otherwise.

Where linear mixed models (LMMs) or multiple linear regression models were employed, the final model was obtained using backward selection (Babyak, 2004). Specifically, starting from the initial model, this involved iteratively removing effects that lacked significance. To ensure robustness, model selection was cross-validated using the Akaike Information Criterion (AIC) (Burnham and Anderson, 2002). The normality and homoscedasticity of conditional residuals of all models were visually checked using the Q-Q plot and the residual-by-predicted plot, respectively. Where adequate, post-hoc pairwise comparisons correcting for multiple testing were performed using Tukey honestly significant difference (HSD) tests.

#### 2.6.1 Group differences in baseline characteristics and MRS quality metrics

Student’s *t*-tests were applied to test differences between groups. If variables were non-normally distributed in at least one group, statistical analysis was conducted using the Wilcoxon Rank-sum test.

#### 2.6.2 Group differences in motor performance

An LMM was used to assess group differences in motor performance (i.e., average scores). The model included score as the dependent variable, with age group (YA and OA), condition (1:1, 1:3, and 3:1), and age group*condition as fixed effects. Participant was included as a random intercept.

#### 2.6.3 Group differences in short-term motor learning

Differences in motor performance (i.e., learning slope) were examined using an LMM with learning slope as the dependent variable. Fixed effects included age group (YA and OA), task variant (1:1, 1:3 and 3:1), and age group*task variant, while participant was added as a random intercept.

#### 2.6.4 Group differences in GABA+, Glx and GSH

Wilcoxon rank-sum tests were used to test for group differences in GABA_pre_, GABA_post_, Glx_pre_ and GSH levels, as these levels were not normally distributed for at least one age group. A t-test was applied to test for group comparisons for the normally distributed GABA_BTT,_ Glx_BTT_, and Glx_post_ levels.

#### 2.6.5 Modulation of GABA+ and Glx levels during task performance and age group differences

Changes in GABA+ and Glx levels during task performance and age group differences in modulation were assessed for both neurometabolites separately using LMMs. GABA+ and Glx, respectively, were added as the dependent variable, and age group (YA and OA), timepoint (pre, BTT, and post), and age group*timepoint as fixed effects. Participant was included as a random intercept.

We also evaluated the Glx/GABA+ ratio at each time point (pre-, during-, and post-task) as an exploratory index of excitatory/inhibitory (E/I) balance. While not used in the main models, these group comparisons are summarized in Supplementary material 4.

To verify that the tissue correction method (and hence the tissue composition) did not bias the results, these analyses were repeated using both CSF-corrected and uncorrected GABA+ values. The results are provided in Supplementary material 5.

#### 2.6.6 Association between motor performance and short-term motor learning and task-related GABA+ modulation, Glx modulation, and resting GSH levels

Separate multiple regression models using automated multiple data imputation to replace missing data points (1 missing GABA_pre_/Glx_pre_ datapoint, and 5 GSH missing datapoints; see 3.1.2 MRS quality metrics) were constructed for two behavioral outcomes, i.e., performance and short-term learning.

To investigate the associations with baseline neurometabolite levels (GABA+, Glx and GSH), each model included fixed effects for age group, GABA_pre_, Glx_pre_, GSH, age group*GABA_pre_, age group*Glx_pre_, age group*GSH, GABA_pre_*Glx_pre_, GABA_pre_*GSH and Glx_pre_*GSH. Similarly, to investigate the relationships with task-related GABA+ and Glx modulation, models included fixed effects for age group, modulation GABA, modulation Glx, group*modulation GABA, age group*modulation Glx, and modulation GABA*modulation Glx. In all models, sex was included as a covariate of no interest.

## 3 Results

### 3.1 Group differences in baseline characteristics and MRS quality metrics

#### 3.1.1 Baseline characteristics

Age groups did not differ with respect to MoCA scores, handedness or any other baseline parameter as shown in Table 1.

#### 3.1.2 MRS quality metrics

A total of 6 out of 200 MRS spectra were excluded from analysis due to either technical issues or poor spectral quality. Specifically, one older adult’s GABA_pre_ and Glx_pre_ scan failed due to a technical error during the first scan session, which could not be repeated, resulting in 1 missing GABA_pre_ and Glx_pre_ measurement. For GSH, 5 spectra were excluded: 3 from older adults [2 due to bad quality (high fit errors exceeding mean + 3 SD), 1 due to unreasonably scaled estimates > median + 5*MAD] and 2 from younger adults [1 due to no detectable GSH signal, 1 due to bas quality (high FWHM exceeding mean + 3 SD)].

The fit error of the GABA+, Glx, and GSH signal, defined as the fit amplitude divided by the standard deviation of the fit residual, full width at half maximum (FWHM; Hz) for GABA+, Glx, GSH and creatine (Cr) and, signal-to-noise ratio (SNR) of GABA+, Glx and, GSH are reported for both groups separately in Table 2.

**Table 2:** Quality measures.

	Younger adults (median±IQR) (mean±SD) ∇	Older adults (median±IQR) (mean±SD) ∇	Group comparison *p*-value of WRST *p*-value of *t*-test ∇
FWHM GABA+	18.79 ± 2.00	19.15 ± 2.21	0.0719
FWHM Glx	14.44 ± 1.44	14.63 ± 2.33	0.0130*
FWHM GSH	15.06 ± 3.39∇	14.58 ± 4.38	0.8559
FWHM Cr	8.66 ± 0.89	9.44 ± 1. 85	<0.0001*
SNR GABA+	12.69 ± 2.04∇	11.24 ± 1.92∇	<0.0001*∇
SNR Glx	15.58 ± 3.24∇	13.87 ± 2.71∇	0.0006*∇
SNR GSH	6.86 ± 1.41∇	7.38 ± 1.60∇	0.2597∇
Fit error GABA+	6.30 ± 1.27∇	6.86 ± 2.01	0.0184*
Fit error Glx	4.80 ± 2.29	5.69 ± 2.91	0.0039*
Fit error GSH	13.15 ± 3.57∇	15.19 ± 5.07∇	0.1248∇

Magnetic resonance spectroscopy (MRS) data quality measures for GABA+, Glx and GSH in younger and older adults. For Glx and GSH, data are pooled over the pre, BTT and post measurement.

Variables denoted by ∇ represent normally distributed data, for which the mean and standard deviation are reported. For non-normally distributed variables, the median and interquartile range are provided. Statistical analyses of non-normally distributed variables were conducted using the Wilcoxon Rank-sum test, while group comparisons between normally distributed variables was analyzed using a Student’s *t*-test. Significant differences are written in bold and denoted with an asterisk.

FWHM, full width at half maximum; GABA, gamma-aminobutyric acid; Glx, glutamate + glutamine; GSH, glutathione; IQR, interquartile range; SD, standard deviation; SNR, signal to noise ratio; WRST, Wilcoxon signed-rank test.

The CSF fraction was higher in older compared to younger adults while there were no significant age group differences in WM or GM fractions (see Table 3).

**Table 3:** Tissue fractions of each VOI per age group.

Metabolite	GM fraction (mean±SD) (median±IQR)∇			WM fraction (mean±SD) (median±IQR)∇			CSF fraction (mean±SD) (median±IQR)∇		
	YA	OA	*p*	YA	OA	p	YA	OA	*p*
GABA+/Glx									
*Pre*	0.65 ± 0.03	0.64 ± 0.03	0.2538	0.33 ± 0.03	0.33 ± 0.04	0.9203	0.01 ± 0.01∇	0.03 ± 0.01	0.0028*
*BTT*	0.65 ± 0.03	0.64 ± 0.02	0.1432	0.33 ± 0.03	0.34 ± 0.03	0.9043	0.01 ± 0.01∇	0.03 ± 0.01	0.0070*
*Post*	0.65 ± 0.03	0.64 ± 0.02	0.1445	0.33 ± 0.03	0.34 ± 0.03	0.9056	0.01 ± 0.01∇	0.03 ± 0.01	0.0074*
GSH	0.65 ± 0.03	0.64 ± 0.03	0.1234	0.33 ± 0.03	0.34 ± 0.03	0.7396	0.01 ± 0.01∇	0.03 ± 0.01	0.0156*

Variables denoted by ∇ represent parameters with non-normal distributions, for which the median and interquartile range (IQR) are reported. These variables were analyzed using a Wilcoxon rank-sum test. Statistical analyses of normally distributed parameters were performed using a pooled *t*-test. Significant group differences are indicated with an asterisk (*) and written in bold.

CSF, cerebrospinal fluid; GSH, glutathione; GABA, gamma aminobutyric acid; GM, grey matter; IQR, inter quartile range; OA, older adults; SD, standard deviation; WM, white matter; YA, younger adults.

### 3.2 Group differences in motor performance & short-term motor learning

#### 3.2.1 Motor performance

There was a significant effect of age group [*F*(1,48) = 37.27, *p* < 0.0001] (OA / YA (mean±SD): 16.52 ± 16.80 / 34.54 ± 21.22), condition [*F*(2,96) = 72.39, *p* < 0.0001], and the age group*condition interaction [*F*(2,96) = 7.67, *p* = 0.0008] on BTT performance (average score S) (see Figure 3).

**Figure 4:**
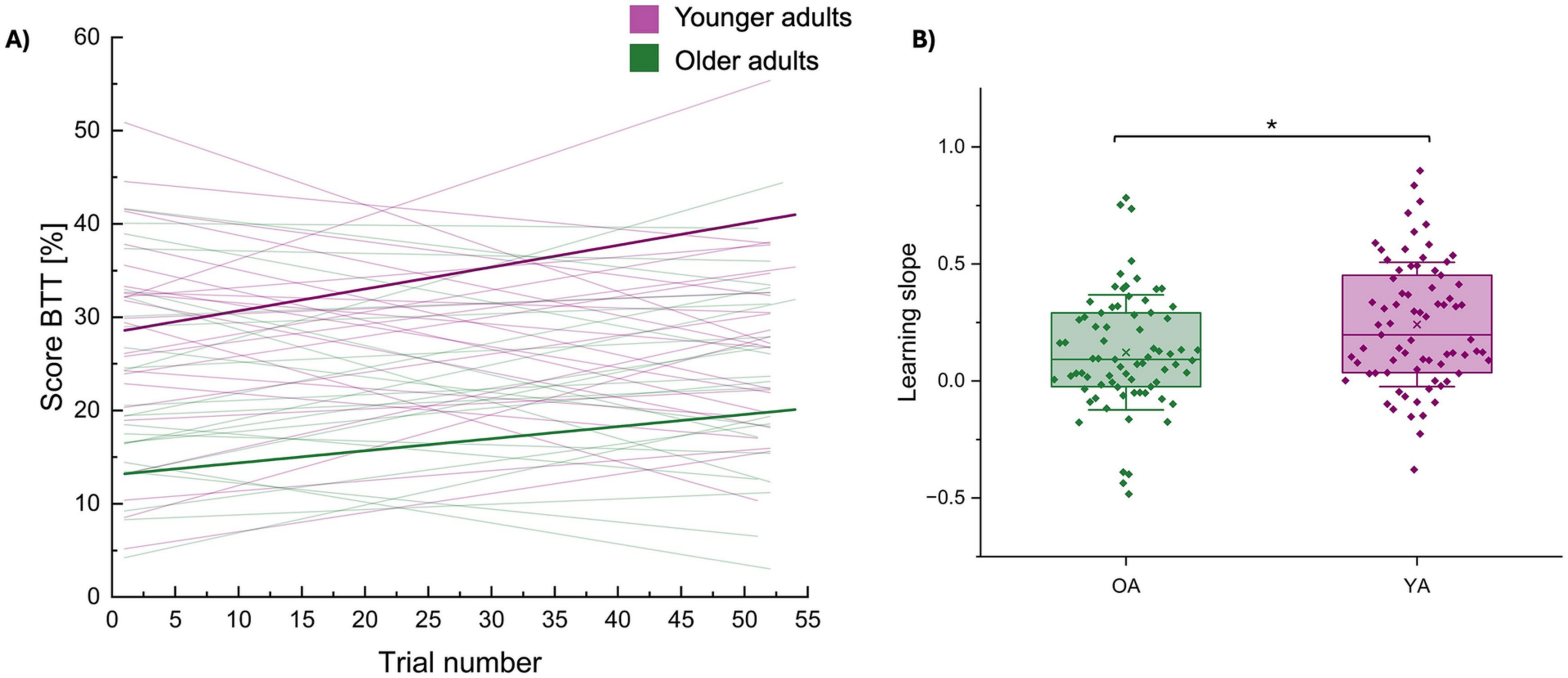
Group differences in short-term motor learning (learning slope). **(A)** Score on the BTT plotted over trial numbers for every participant separately (younger adults in purple; older adults in green). Short-term motor learning was quantified using the slope of these linear fits, corresponding with Hehl et al. (2025). Individual learning slopes are shown as thin purple or green lines for younger and older adults, respectively. Bold lines represent the group averages. **(B)** The average learning slopes per age group are represented by a boxplot (percentile 25–75) ± 1 SD (whiskers), the line represents the median and individual datapoints are superimposed. Younger adults demonstrated a steeper learning progress (i.e., making bigger improvements from trial to trial) than older adults. * represents a statistical difference of *p* < 0.05. BTT, bimanual tracking task; OA, older adults; YA, younger adults.

Post-hoc pairwise comparisons (Table 4) showed a consistent difference in performance, with older adults consistently performing worse compared to younger adults in all conditions [1:1: t(96) = −22.61, *p* < 0.0001; 1:3: t(96) = −17.72, *p* < 0.0001; 3:1: t(96) = −13.60, *p* = 0.0008]. In both age groups, the 1:1 condition was consistently better executed than the 1:3 condition [OA: t(96) = 8.58, *p* < 0.0001; YA: t(96) = 13.47, *p* < 0.0001], and the 3:1 condition [OA: t(96) = 8.27, *p* < 0.0001; YA: t(96) = 17.29, *p* < 0.0001]. Finally, there was no difference in the execution between the 1:3 and the 3:1 condition [OA: t(96) = −0.30, *p* = 1.0000; YA: t(96) = 3.82, *p* = 0.1871].

**Table 4:** Tukey HSD all pairwise comparison analysis of the age group*condition effect on BTT score.

Age group	Condition	-Age group	-Condition	Difference	T Ratio	Lower 95% CI	Upper 95% CI	*p*-value
OA	1:1	OA	1:3	8.58	5.27	3.84	13.31	<0.0001*
OA	1:1	OA	3:1	8.27	5.08	3.54	13.01	<0.0001*
OA	1:1	YA	1:1	−22.61	−7.00	−32.01	−13.22	<0.0001*
OA	1:3	OA	3:1	−0.30	−0.19	−5.04	4.43	1.0000
OA	1:3	YA	1:3	−17.72	−22.61	−27.12	−8.33	<0.0001*
OA	3:1	YA	3:1	−13.60	−4.21	−23.00	−4.21	0.0008*
YA	1:1	YA	1:3	13.47	8.27	8.73	18.21	<0.0001*
YA	1:1	YA	3:1	17.29	10.62	12.55	22.02	<0.0001*
YA	1:3	YA	3:1	3.82	2.34	−0.92	8.55	0.1871

Significant *p*-values are written in bold and indicated with an asterisk.

CI, confidence interval; OA, older adults; YA, younger adults.

#### 3.2.2 Short-term motor learning

There was a significant effect of age group [*F*(1,48) = 6.07, *p* = 0.0174) (OA / YA (mean±SD): 0.12 ± 0.25 / 0.24 ± 0.27] on short-term motor learning (slope) (see Figure 4), with younger adults demonstrating a steeper learning progress (i.e., making bigger improvements from trial to trial). None of the other effects were significant (all, *p* > 0.05).

### 3.3 Group differences in GABA+, Glx and GSH

#### 3.3.1 GABA+

**Figure 5:**
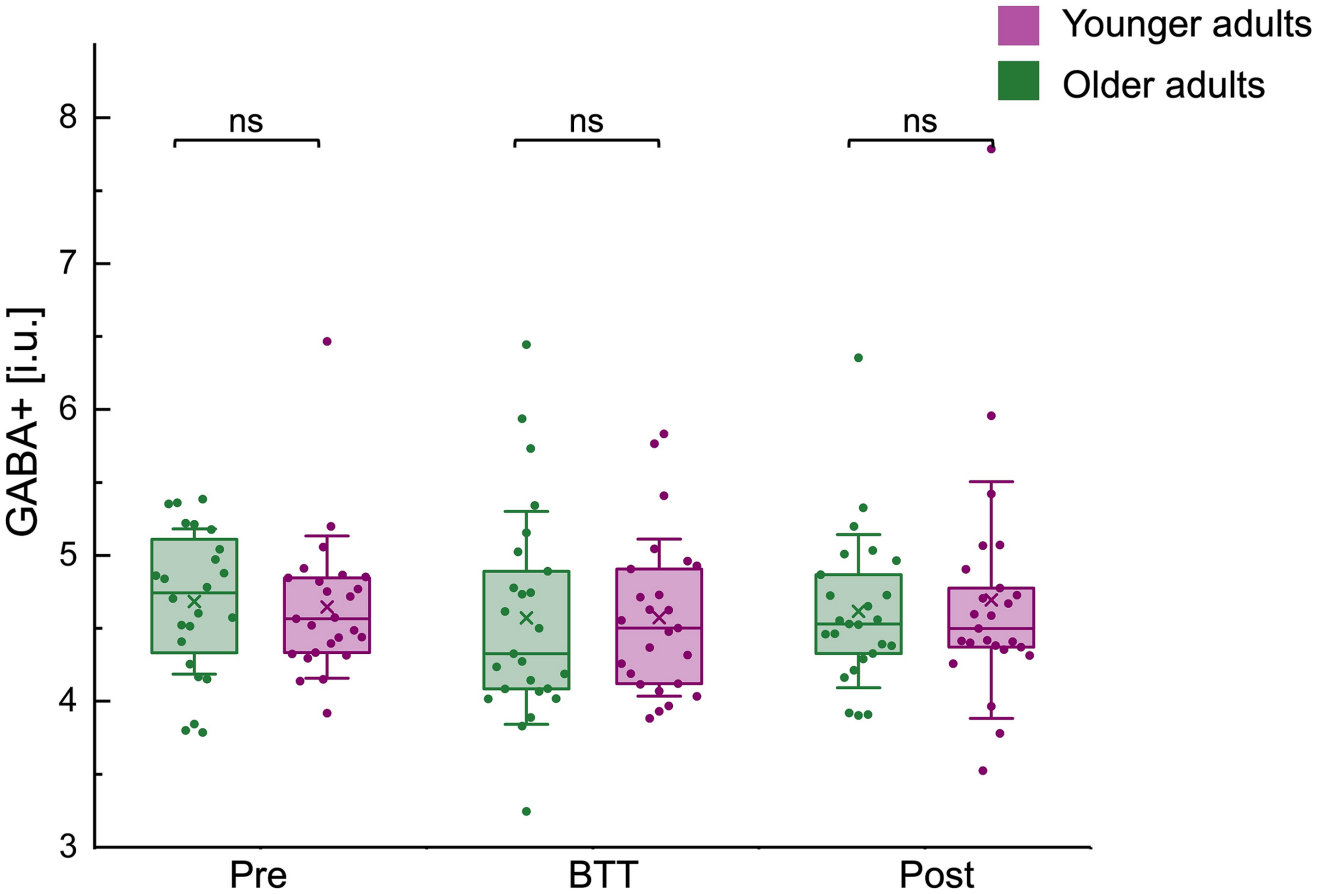
GABA+ (i.u.) levels before (pre), during (BTT), and after (post) task performance for both age groups. Boxplots (box = percentile 25–75, whiskers = ±1 SD, line = median, × = mean) with individual datapoints superimposed. No significant differences in GABA+ levels were observed between age groups across all time points. BTT, bimanual tracking task; GABA, gamma-aminobutyric acid; i.u., institutional units; ns, non-significant.

No significant differences in GABA+ levels were observed between age groups across all time points: pre-BTT GABA levels (GABA_pre_ OA / YA (median±IQR): 4.74 ± 0.85 / 4.56 ± 0.52; z = 0.89, *p* = 0.3735), BTT GABA levels (GABA_BTT_ OA / YA (mean±SD): 4.57 ± 0.73 / 4.57 ± 0.54; t(48) = 0.01, *p* = 0.9958), and post-BTT GABA levels (GABA_post_ OA / YA (median±IQR): 4.53 ± 0.61 / 4.50 ± 0.48; z = 0.06, *p* = 0.9536) (Figure 5).

#### 3.3.2 Glx

**Figure 6:**
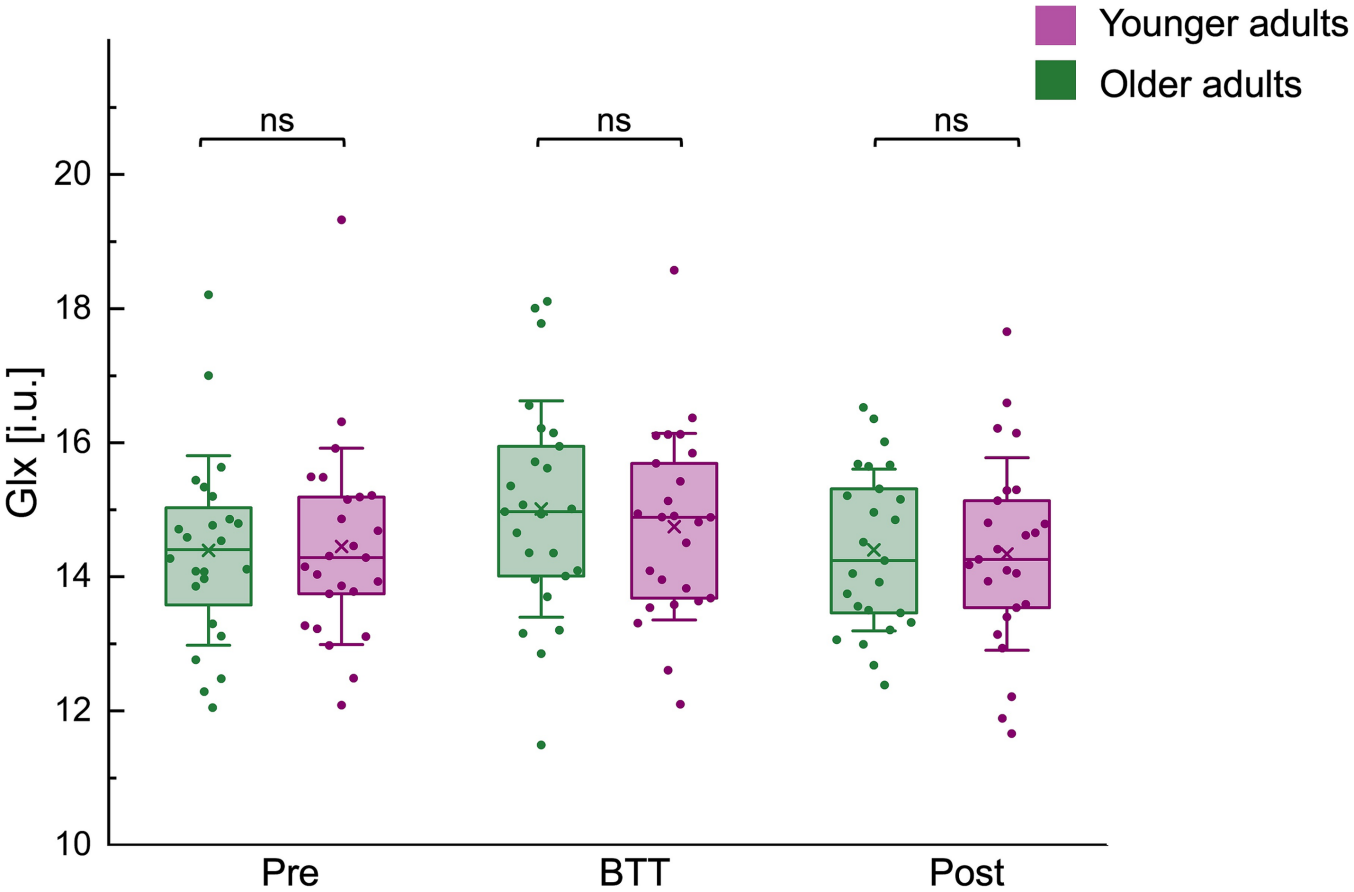
Glx (i.u.) levels before, during, and after task performance for both age groups. Glx (i.u.) levels before (pre), during (BTT), and after (post) performance of the bimanual tracking tasks (BTT) for both age groups are represented by a boxplot (box = percentile 25–75, whiskers = ±1 SD, line = median, × = mean), individual datapoints are superimposed. No significant differences in Glx levels were observed between age groups across all time points. BTT, bimanual tracking task; Glx, combined measure for glutamine and glutamate; i.u., institutional units; ns, non-significant.

No significant differences in Glx levels were found between age groups across all time points: pre-BTT Glx levels [Glx_pre_ OA / YA (median±IQR): 14.40 ± 1.67 / 14.29 ± 1.69; z = 0.00, *p* = 1.0000], BTT Glx levels [Glx_BTT_ OA / YA (mean±SD): 15.01 ± 1.62 / 14.75 ± 1.39; t(48) = −0.62, *p* = 0.5384], and post-BTT Glx levels [Glx_post_ OA / YA (mean±SD): 14.40 ± 1.21 / 14.34 ± 1.44; t(48) = −0.16, *p* = 0.8714] (Figure 6).

#### 3.3.3 GSH

**Figure 7:**
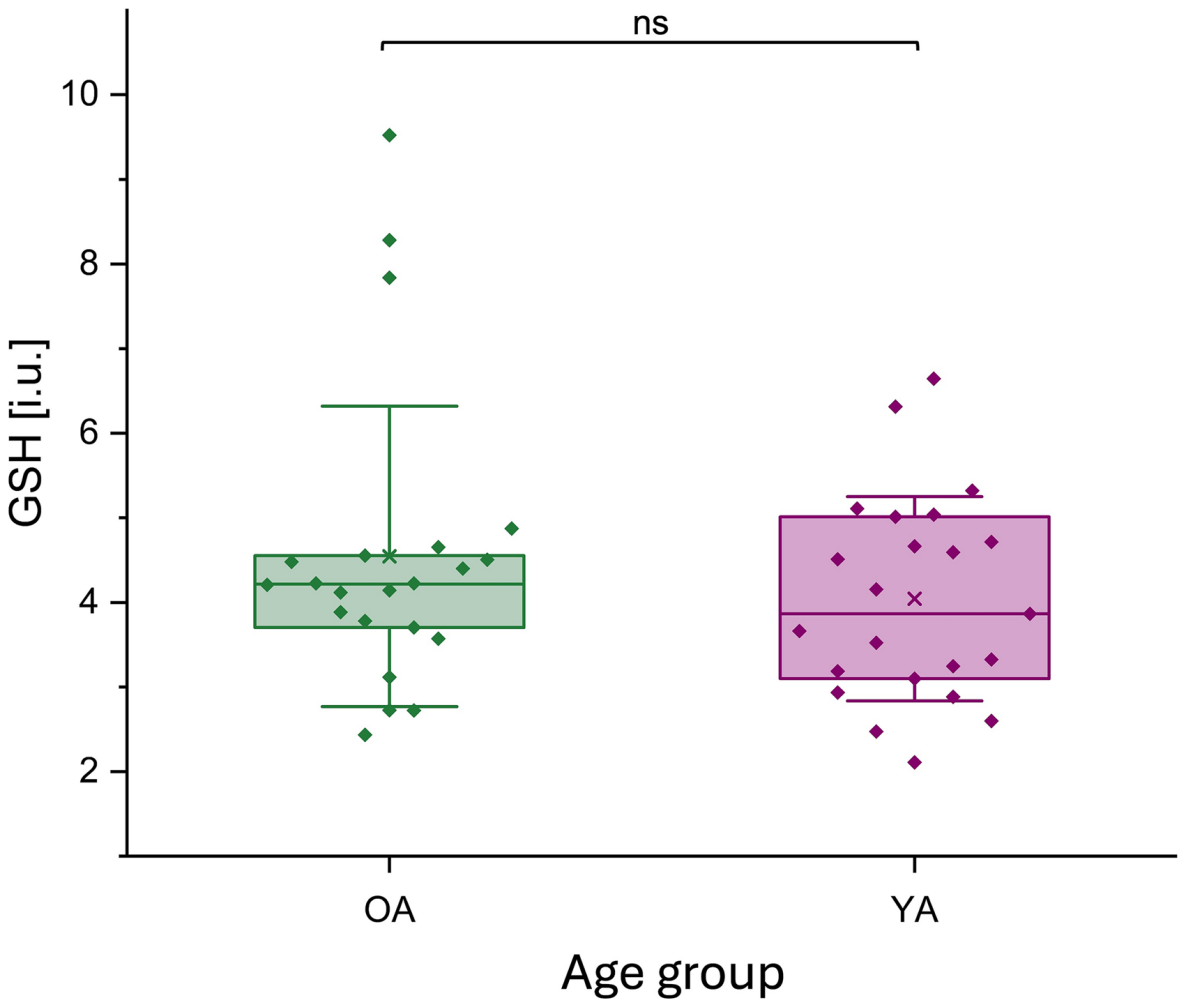
GSH (i.u.) concentration for younger and older adults. GSH levels (i.u.) are represented by a boxplot (box = percentile 25–75, whiskers = ±1 SD, line = median, × = mean), datapoints are superimposed, and a violin plot for young and older adults separately. No significant differences in GSH levels were observed between age groups. GSH, glutathione; i.u., institutional units; ns, non-significant; OA, older adults; YA, younger adults.

There was no significant difference in GSH levels between both age groups (GSH OA / YA (median±IQR): 4.22 ± 0.91 / 3.87 ± 1.91; *z* = 0.47, *p* = 0.6416) (Figure 7).

### 3.4 Modulation of GABA+ and Glx levels during task performance and group differences

The GABA_post_ data of one young adult was excluded from the analysis because its residual represented a visual outlier on the Q-Q plot that could not be accounted for using data transformation. This was corroborated using JMP’s ‘Robust Fit Outliers’ method based on Huber’s M-estimation (*K* = 4).

There were no significant effects of timepoint (pre, BTT and post levels), suggesting that GABA+ and Glx levels did not modulate over time at group level. Furthermore, there was no age group effect and no age group*timepoint interaction (all, *p* > 0.05).

### 3.5 Association between baseline neurometabolite levels, task-related GABA modulation, Glx modulation, and motor performance and short-term motor learning

#### 3.5.1 Baseline neurometabolite levels

##### 3.5.1.1 Performance

Two younger male participants were excluded from the analysis due to their residuals being outliers, based on externally studentized residuals exceeding ±2 (Belsley et al., 1980), which disrupted the normality of the residual distribution.

Neither baseline GABA+, Glx, nor GSH levels predicted motor performance. In particular, motor performance [*F*(2, 47) = 34.31, *p* < 0.0001, *R*^2^ = 0.60] was only significantly predicted by age group [*β* = −0.74, t(1) = −1.28, *p* < 0.0001], with younger adults performing better as compared to older adults (as reported in 3.2.1 Motor performance). Sex [*β* = −0.26, t(1) = −1.28, *p* = 0.0085], a covariate of no interest, was a significant predictor too, with males performing better as compared to females [Performance M / F (mean±SD): 29.25 ± 13.38 / 23.11 ± 13.68]. No other predictors contributed significantly to the model (all, *p* < 0.05). See Supplementary Tables 3–6 for the parameter estimates and effect tests of both the full and final model.

##### 3.5.1.2 Short-term motor learning

Task-related modulation of GABA+ and Glx did not significantly predict motor performance. Specifically, the final model was not significant [*F*(2, 49) = 2.68, *p* = 0.08, R^2^ = 0.10]. Parameter estimates and effect tests of both the full and final model are provided in Supplementary Tables 7–10.

#### 3.5.2 GABA+ and Glx modulation

Neither GABA+ nor Glx modulation significantly predicted performance and short-term motor learning. For both models, only age group was a significant predictor (performance: *β* = −0.66, t(1) = −6.22, *p* < 0.0001 / short-term motor learning *β* = −0.31, t(1) = −2.26, *p* = 0.0287), with younger adults performing better and demonstrating steeper learning slopes (as reported in section 3.2.1 Motor performance and 3.2.2 Short-term motor learning), respectively. Full model estimates are provided in Supplementary material 6.

## 4 Discussion

Firstly, and in line with our expectations, younger adults outperformed older adults in both the simple and complex conditions of the bimanual task. Specifically, they demonstrated superior overall performance and exhibited greater short-term motor learning. Secondly, we could not demonstrate significant baseline differences in cerebellar GABA+, Glx, or GSH levels between both age groups. In addition, neither did GABA+ or Glx levels show task-related modulation at the group level as indicated by the lack of differences over time (GABA_pre_, GABA_BTT_, and GABA_post_ as well as Glx_pre_, Glx_BTT_, and Glx_post_), nor was there an age-group effect on modulation. Finally, motor performance and short-term motor learning were predicted solely by age group, replicating the behavioral results, but neither baseline cerebellar GABA+, Glx or GSH levels nor task-related modulation of cerebellar GABA+ or Glx levels.

### 4.1 Younger adults excel in motor performance and short-term motor learning

Bimanual motor performance was better in younger as compared to older adults. As expected, motor performance was better in the simpler (iso-frequency) as compared to the more complex (non-iso-frequency) task variants. Additionally, performance differences between age groups were greater in the iso-frequency compared to the non-isofrequency variant, favoring the younger group. These findings are consistent with previous research (Swinnen and Wenderoth, 2004; Boisgontier et al., 2018; Maes et al., 2022; Van Hoornweder et al., 2022; Van Ruitenbeek et al., 2023).

In addition, the short-term motor learning rate was significantly higher in younger as compared to older adults, consistent with previous literature (e.g., Swinnen, 1998; Shea et al., 2006; for a review see: Voelcker-Rehage, 2008). Nevertheless, although motor performance was generally lower in older than younger adults, older adults still retain the ability to acquire new motor skills, albeit at a slower rate than younger adults (Raz et al., 2000; Smith et al., 2005; Voelcker-Rehage, 2008; Gooijers et al., 2024). This ability is mediated by task complexity, among others, with younger adults showing significantly faster improvement as task difficulty increases (Voelcker-Rehage, 2008). Hence, the lower learning rate in older as compared to younger adults observed in this study might be attributable to the overall complexity of the bimanual tracking task, encompassing both the simple and complex non-iso-frequency conditions.

### 4.2 No age-related difference in cerebellar GABA+, Glx and GSH levels

At the cortical level, previous studies demonstrated reduced GABA+ levels in older adults as compared to younger adults (e.g., Porges et al., 2017a; Chalavi et al., 2018; Cassady et al., 2019; Chamberlain et al., 2021; Dobri and Ross, 2021; Maes et al., 2021; Heise et al., 2022), but findings remain inconsistent, as other studies reported no significant differences between age groups (e.g., Chalavi et al., 2018; Hermans et al., 2018b; Maes et al., 2018; Cuypers et al., 2021). It is important to note that different brain regions were studied, i.e., mostly cortical regions and especially the SM1 and primary sensory cortices (visual, auditory, somatosensory) (Li et al., 2022). To the best of our knowledge, this study is the first to examine age-related differences in neurometabolite levels in the cerebellum using MRS, and we found no significant age-related differences in cerebellar GABA+ concentration between both age groups. Interestingly, despite an increased CSF fraction and a trend toward lower GM in the voxels of older compared to younger adults, uncorrected GABA+ levels did not differ between age groups either (see Supplementary material 5). This contrasts with some studies that suggest that age-related differences in GABA+ levels (e.g., in the frontal cortex and SM1 cortex) are driven by bulk tissue changes and thus depend on the tissue correction method used (Porges et al., 2017b; Maes et al., 2018). It has been suggested that age differences in GABA+ are region-specific, potentially emerging in certain cortical areas while being absent or emerging later in others (Hermans et al., 2018a; Maes et al., 2021; Li et al., 2022). The latter may also be the case in the cerebellum.

In line with Ding et al. (2016) and Maghsudi et al. (2020), both lifespan studies using whole brain magnetic resonance spectroscopy imaging (MRSI), we found no age-related differences in cerebellar Glx levels. This aligns with some studies looking at cortical Glx (e.g., Mooney et al., 2017; Simmonite et al., 2019) but contradicts others that did reported age-related declines in cortical regions (e.g., Levin et al., 2019). Similar to GABA+, it has been suggested that age-related differences in Glx may be region-specific (Kaiser et al., 2005; Marjańska et al., 2017), and hence might evolve differently in the cerebellum as compared to cortical areas.

The finding that GABA+ and Glx levels did not differ between younger and older adults aligns with a recent transcranial magnetic stimulation (TMS) study (Van Malderen et al., 2025) examining CBI. CBI is thought to reflect the GABA-mediated inhibitory influence exerted from the cerebellar cortex to the dentate nucleus and motor cortex via the dentate-thalamo-cortical pathway (Ugawa et al., 1995; Fernandez et al., 2018). Similar to the current results, no age-related differences in CBI were demonstrated. While the exact relationship between MRS and TMS measures remains unclear—TMS primarily captures receptor-level functional aspects of GABAergic and glutamatergic neurotransmission (i.e., fast-acting phasic inhibition), whereas MRS predominantly reflects broader neurometabolic pools related to tonic inhibition (Stagg et al., 2011b; Rae, 2014; Cuypers and Marsman, 2021)—the two methods are considered complementary in assessing glutamergic and GABAergic neurotransmitter function (Stagg et al., 2011a; Tremblay et al., 2013; Cuypers and Marsman, 2021). These complementary findings support the idea that there may indeed be no significant age-related differences in cerebellar GABA+ and Glx functioning at the group level.

Our findings indicated no age-related differences in GSH levels in the right cerebellar hemisphere, supporting the notion that cerebellar GSH levels may remain stable across aging. This aligns with a recent systematic review suggesting that age-related GSH alterations are region-specific, with the cerebellum, among other regions, showing relative stability (Detcheverry et al., 2023).

Given that GSH plays a critical role in buffering reactive oxygen species (ROS)—associated with oxidative stress in high levels— its stability in the cerebellum may reflect this region’s relative resistance to aging (Schieber and Chandel, 2014). Supporting this idea, post-mortem studies have shown that, among others, the cerebellum exhibits fewer mitochondrial DNA deletions (Corral-Debrinski et al., 1992), reduced oxidative damage (Mecocci et al., 1993; Venkateshappa et al., 2012), less age-related DNA methylation compared to cortical regions (Horvath et al., 2015). This resilience may be linked to the cerebellum’s inherently low regional glucose metabolism, fairly low aerobic glycolysis, and minimal age-related change in glucose metabolism—factors that may lower susceptibility to oxidative stress (Bentourkia et al., 2000; Vaishnavi et al., 2010; Goyal et al., 2014).

Oxidative stress, driven by increased ROS production, an inevitable byproduct of oxygen metabolism in aerobic cells—is a key factor in aging (Ames et al., 1993). GSH plays a crucial role in buffering these ROS (Dringen et al., 2000). Evidence from rodent studies suggests that GSH homeostasis becomes increasingly disrupted with age, with several reports indicating lower GSH concentrations in older animals (e.g., Chen et al., 1989; Maher, 2005; Zhu et al., 2006; Rebrin et al., 2007).

Findings in humans, however, are more mixed and vary by region. Specifically, peripheral studies investigating blood GSH levels report declining GSH levels with age (Yang et al., 1995; Jones et al., 2002; Giustarini et al., 2006), while postmortem analyses yield contrasting results, showing either no age-related differences in GSH levels in the frontal cortex and cerebellum (Venkateshappa et al., 2012), or even increases in the caudate, frontal cortex, and cerebellum with advancing age (Tong et al., 2016). *In vivo*, studies are similarly inconsistent, with reports of stable (Gong et al., 2022) or lower GSH levels in older adults, for example, in the occipital and parietal lobes (Emir et al., 2011), precuneus, as well as in the posterior cingulate cortex (Suri et al., 2017). Conversely, other studies found higher GSH levels in the medial frontal and sensorimotor cortices in older adults after accounting for age-related atrophy (Hupfeld et al., 2021). Taken together, previous studies remain inconclusive, however, they emphasize the region-specific nature of age-related GSH changes. Our results contribute to this body of literature by reinforcing the idea that the cerebellum may be particularly resistant to GSH depletion with aging.

The preservation of GABA+, Glx, and GSH levels in the aged cerebellum may be attributed to its relatively slower aging process as compared to other (cortical) brain areas, potentially related to the so-called cerebellar reserve (Liang and Carlson, 2020; Arleo et al., 2024). While studies show age-related declines in cerebellar volume and corresponding reductions in motor performance (Jernigan et al., 2001; e.g., Bernard and Seidler, 2013; Bernard and Seidler, 2014), genetic and epigenetic evidence suggests that the cerebellum undergoes slower aging compared to other brain regions and may potentially be more resistant to the development of pathological markers of neurodegeneration (Liang and Carlson, 2020; Arleo et al., 2024). This resilience has been suggested to be due to the cerebellum’s greater epigenetic stability (Horvath et al., 2015), fewer age-related deletions in mitochondrial DNA compared to the cortex (Corral-Debrinski et al., 1992), and lower levels of oxidative damage to both mitochondrial and nuclear DNA (Mecocci et al., 1993). Therefore, while the average age of older adults in our sample was comparable to that of studies showing neurometabolic differences in cortical areas (e.g., Chalavi et al., 2018; Chamberlain et al., 2021; Maes et al., 2021), it is possible that they were not yet old enough to show detectable neurometabolic changes in the cerebellum. In addition, the older adults in our study were physically active, which may protect against metabolic decline, as physical activity is known to have a protective effect on the brain and may help preserve overall brain integrity (Boisgontier et al., 2018) and neurometabolite levels [GABA+ and Glx (Bütefisch et al., 2000; Maddock et al., 2016) as well as GSH (Rai et al., 2018)]. Future studies could consider including a less active, older group (e.g., >80 years) to further investigate the potential impact of age and physical activity on neurometabolite levels in very old age.

Alternatively, while age-related differences in neurometabolite levels may exist, they may have fallen below the detection threshold of our methodology. Specifically, just as different parts of the cerebellum undergo atrophic changes to varying degrees (Andersen et al., 2003; Paul et al., 2009), it is possible that specific metabolite changes occur on a smaller scale within functional cerebellar subregions. However, these subtle differences may be lost within the broader cerebellar voxel, which encompasses nearly the entire right cerebellar hemisphere and thus has limited spatial resolution. In addition, it could be that a longitudinal study is needed to detect such subtle differences and account for individual variability. Indeed, Zuppichini et al. (2024) demonstrated GABA differences in the right auditory, bilateral sensorimotor and ventrovisual regions between young and older adults in a longitudinal analysis, whereas no differences were observed in a cross-sectional comparison. Finally, it is important to consider the contribution of macromolecules to the GABA signal. Given that older exhibit higher macromolecule levels as compared to younger adults (Aufhaus et al., 2013; Marjańska et al., 2018), potential age-related differences in GABA itself (without macromolecules) may be underestimated.

### 4.3 No modulation of cerebellar GABA+ and Glx levels during task performance as compared to baseline and no group differences in modulation

GABA and Glx concentrations in a specific brain region may exhibit rapid modulation—either increasing or decreasing—as a function of task performance or motor learning, depending on the region’s precise role in the process being examined (e.g., Pasanta et al., 2023; Li et al., 2024b). Despite the increased interest in the modulation of GABA and Glx levels recently, evidence is still scarce, with only a few studies investigating visuomotor coordination learning tasks and just one study specifically focusing on the cerebellum (Nettekoven et al., 2022). Some studies reported GABA reductions in SM1 during motor performance and learning, which were linked to enhanced motor learning and improved visuomotor task performance (Floyer-Lea et al., 2006; Kolasinski et al., 2019; Maes et al., 2022) while others reported an increase in Glx which was correlated to motor actions but did not predict visual performance (Kurcyus et al., 2018; Volovyk and Tal, 2020). Nevertheless, findings are inconsistent, with considerable variability in effect sizes and directionality for GABA levels (Pasanta et al., 2023). Furthermore, prior research has shown that neurometabolite changes are highly region-specific, with some studies failing to detect modulation in certain areas while observing significant effects in others (Chalavi et al., 2018; Coxon et al., 2018; Frangou et al., 2019). Finally, specific features of the task also seem to influence temporal neurometabolite changes (Chalavi et al., 2018; Hehl et al., 2025).

In the present study, we did neither observe task-related modulation of GABA+ or Glx levels at the group level, nor did we find age-related differences in the modulation of these neurometabolites in the cerebellum. These findings align with previous studies that report mixed evidence for GABA and Glx modulation depending on the specific task and brain region, and might point to the absence of a modulation of GABA and Glx in the context of bimanual coordination learning in the cerebellum. Alternatively, it could be that transient changes in neurometabolite levels within a specific cerebellar region occur on a small scale within a specific functional sub-region. Indeed, prior work using magnetic resonance spectroscopic imaging (MRSI) has demonstrated GABA changes in the dentate nucleus but not in the cerebellar hemisphere (Nettekoven et al., 2022), suggesting that modulation might occur at a finer spatial scale than our voxel, which encompassed most of the right cerebellar hemisphere, could capture. It should be noted that the cerebellum is composed of a mosaic of functional subregions, each specialized for distinct roles ranging from fine motor control to social and linguistic processing (King et al., 2019; Nettekoven et al., 2024). As such, our cerebellar voxel likely captured a broad and heterogeneous area, in contrast to cortical MRS studies that often target more functionally discrete and homogeneous regions. Alternatively, modulation might take place on an even smaller scale, given the presence of multiple pathways underlying synaptic long-term potentiation (LTP) in the cerebellum (Binda et al., 2016) and other forms of cerebellar plasticity occurring at microscale (Schonewille et al., 2011). Due to the relatively large voxel in MRS, and hence, limited spatial resolution, averaging across multiple layers, subregions and functional domains—each serving distinct sensorimotor, cognitive, or affective functions—subtle, region-specific neurometabolic differences might go undetected. Future studies employing higher spatial resolution and region-specific targeted approaches may provide further insights into the subtle dynamics of neurometabolite regulation in the cerebellum.

Along the same lines, our use of 11-min task blocks may have limited our ability to detect modulation of metabolite levels. On one hand, dynamic fluctuations may have occurred outside the temporal sensitivity of our block-averaged approach, potentially masking transient changes (Bell et al., 2023). On the other hand, it is also possible that the duration of task engagement was insufficient for eliciting measurable neurometabolic changes, as longer or more intensive task practice might be required for such effects to emerge. The absence of detectable GABA+ or Glx modulation in our study may therefore reflect limitations in temporal resolution rather than a true absence of change. Future studies could benefit from exploring sliding-window analyses to capture transient modulations that are otherwise obscured by long block durations with a block-averaged analysis (Chen et al., 2017; Bell et al., 2023) as well as prolonged task paradigms to test for delayed or cumulative effects.

### 4.4 No association between motor performance and short-term motor learning and task-related GABA+ modulation, Glx modulation, and baseline neurometabolite levels

Since we found that neither baseline GABA+, Glx and GSH levels, nor GABA+ and Glx modulation predicted motor performance or short-term motor learning at a group-level, it may be that cerebellar neurometabolite concentrations remain relatively stable and not directly relate to age-related differences in behavioral outcomes. Instead, their effects could be mediated by other age-related factors influencing cerebellar function. For example, declines in norepinephrine levels may weaken GABAergic signaling (Watson and McElligott, 1983; Heron et al., 1996; Zhang et al., 2010), while age-related changes in Purkinje cell electrophysiology (Rogers et al., 1981; Chung et al., 2003) and synaptic signaling (Takahashi et al., 2009) could also play a role. A detailed investigation of these mechanisms is, however, beyond the scope of the current study.

It is also plausible that age-related differences in behavior and motor learning are not primarily driven by age-related changes in cerebellar neurometabolite levels but rather by broader alterations in cerebellar morphology, and both functional and structural connectivity. Indeed, Bernard and Seidler (2014) have argued that reductions in cerebellar volume, and particularly diminished connectivity with cortical and subcortical regions, may contribute significantly to age-related motor deterioration. To further clarify the complexity of cerebellar contributions to motor performance in aging, future studies may benefit from integrating morphometric analyses (e.g., voxel-based morphometry) with neurometabolic, connectivity (diffusion-weighted imaging and functional magnetic resonance imaging), and behavioral data. Such multimodal approaches could help disentangle whether structural, functional, and neurochemical differences interact to mediate motor outcomes in older adults.

Moreover, as already discussed at length in the above sections, it might be that the neurometabolite levels in specific functional sub-regions rather than the cerebellar hemisphere as a whole are linked to behavioral outcome. Indeed, while Nettekoven et al. (2022) did not observe a group-level reduction in GABA during an adaptation task using MRSI, they reported that greater early adaptation-driven GABA decreases in the right dentate nucleus were associated with better performance. Due to the relatively large voxel size in our study, we were unable to differentiate between cerebellar subregions such as the hemisphere and the deep nuclei, which may have masked subtle but functionally relevant differences.

Finally, it is important to consider that bimanual coordination relies on a distributed network of regions beyond the cerebellum, including—but not limited to— primary motor (M1) and sensory (S1) cortices, supplementary motor area (SMA), premotor cortex, cingulate motor area, basal ganglia (Laplane et al., 1977; e.g., Brinkman, 1981; Brinkman, 1984; Tanji et al., 1987; Tracy et al., 2001; Debaere et al., 2004; Swinnen and Wenderoth, 2004; Jantzen et al., 2009). Indeed, age-related atrophy and alterations in functional and structural connectivity within this network have been shown to contribute to impaired bimanual coordination (e.g., Goble et al., 2010; Solesio-Jofre et al., 2014; Serbruyns et al., 2015; Fujiyama et al., 2016; Serbruyns et al., 2016). It might therefore be interesting if future work would use a network-level approach to better understand how aging affects the complex interplay between cerebellar, other subcortical and cortical regions involved in motor control and learning.

### 4.5 Considerations, limitations and future perspectives

Most of the limitations of the study have been mentioned earlier, but for clarity, a summary is provided here.

First, it is of importance to acknowledge the contribution of macromolecules to the GABA+ signal. Specifically, since macromolecule levels are higher in older as compared to younger adults (Aufhaus et al., 2013; Marjańska et al., 2018; Genovese et al., 2024), potential age-related differences in GABA+ may be under-estimated. Moreover, the use of macromolecule-suppressed GABA measurements may enhance the relationship between GABA levels and motor performance (Mikkelsen et al., 2018), put still poses several methodological difficulties.

Second, Glx is a compound measure including both glutamate and glutamine. Its measurement as a single entity may lead to different age-related effects than assessing glutamate and glutamine independently (Hädel et al., 2013; Roalf et al., 2020). Specifically, in certain cortical brain regions, glutamate has been shown to decline with age, whereas glutamine demonstrated an increase (Hädel et al., 2013). Due to the considerable spectral overlap between their resonances, higher magnetic field strengths are recommended as they will improve separation, enabling more precise quantification of both metabolites (Hetherington et al., 1997). Furthermore, other MRS sequences—such as PRESS or STEAM—are better suited for quantifying Glx than the difference spectrum derived from the GABA-optimized MEGA-PRESS sequence used in this study (Maddock et al., 2018).

Third, neurometabolite levels were measured within a relatively large voxel to ensure an adequate signal-to-noise ratio. However, as previously discussed, age-related changes and modulation of neurometabolite concentrations might occur on a much smaller spatial scale, which is particularly relevant for the cerebellum, given its high neuronal density and multiple functionally distinct subregions (King et al., 2019; Nettekoven et al., 2024).

Finally, it is important to interpret comparisons between this study and previous research with caution, as substantial methodological differences exist across different MRS studies [e.g., edited vs. unedited MRS, voxel size, and TE–which may be suboptimal in HERMES and HERCULES scans when quantifying multiple metabolites simultaneously (Detcheverry et al., 2023)].

Future research using a higher resolution of MRS—using higher field strengths (7 T) —should address age-related differences in baseline neurometabolite levels and their potential modulation in the context of motor performance and learning on a smaller scale.

## 5 Conclusion

Our findings indicate superior motor performance and faster short-term motor learning in younger compared to older adults. However, no significant age-related differences were observed in cerebellar GABA+, Glx, or GSH levels, nor did GABA+ or Glx concentrations modulate during task performance. These results align with previous research suggesting that neurometabolite alterations in aging are region-specific, with the cerebellum being potentially more resilient to neurometabolic aging. Since neither baseline GABA+, Glx, or GSH levels nor task-related modulation of GABA+ and Glx predicted motor performance or learning, cerebellar neurometabolite concentrations may not directly underlie age-related behavioral changes. Instead, volumetric decline and alterations in structural and functional connectivity in the cerebellum, as well as alterations in other brain regions in the network underlying bimanual motor control, may play a more prominent role. While our findings do not support a direct role of cerebellar neurometabolite levels in age-related motor performance differences, they underscore the complexity of neurochemical aging.

## Data Availability

The raw data supporting the conclusions of this article will be made available by the authors, without undue reservation.
