# Supplementary material for: GABA, Glx, and GSH in the cerebellum: their role in motor performance and learning across age groups
Source: Front Aging Neurosci. 2025 Jul 3;17:1626417. doi: 10.3389/fnagi.2025.1626417 (PMC12267199; doi:10.3389/fnagi.2025.1626417)
Supplement: Supplementary file 1 [file Data_Sheet_1.docx]

Supplementary Material

# Supplement 1. Bimanual tracking task (BTT)


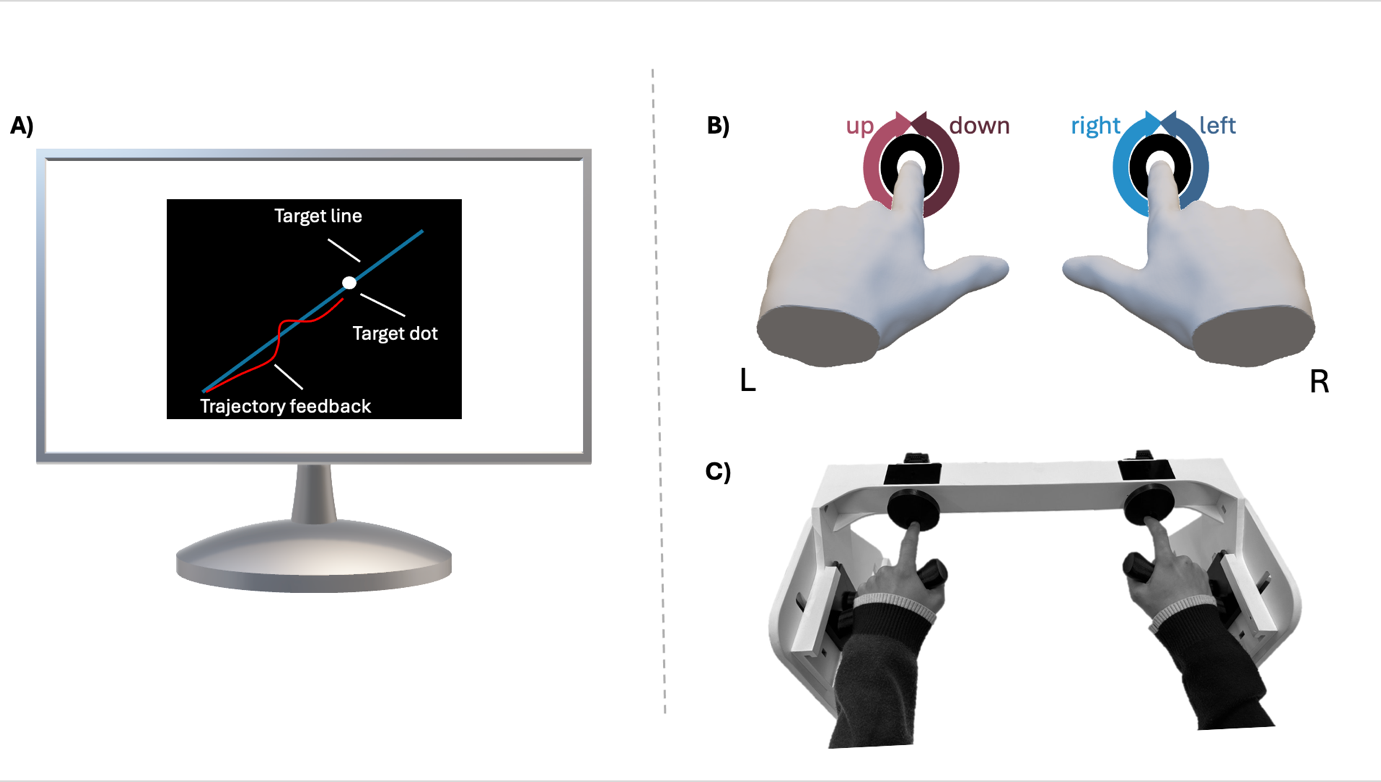


***Supplemental Figure 1.*** *Overview of the bimanual tracking task (BTT).* ***A)*** *Each trial began with a blue target line on a black background that remained visible until the end of each trial. The task required participants to track a white target dot as it moved at a constant speed along a blue target line. A red line visualized the real-time trajectory of the cursor (i.e., trajectory feedback).* ***B)*** *The right hand coordinated horizontal movements (i.e., right / left) with turning right/clockwise resulting in rightward movement. The left hand controlled vertical movements (i.e., up / down) with turning right/clockwise resulting in upward movement).* ***C)*** *Each hand was placed on a handle, with the index fingers positioned in the circular grooves of rotatable dials. While maintaining contact with the handles, only the index fingers could move.*

The task required participants to track a white target dot as it moved at a constant speed along a blue target line (as visualized in supplemental Figure 1.A). Using coordinated movements of both index fingers, participants had to match the dot’s trajectory as accurately as possible. Specifically, the right hand coordinates horizontal movements (right / left; turning right/clockwise results in rightward movement), while the left hand controls the vertical movements (up / down; turning right/clockwise results in upward movement) (Supplemental Figure 1.B). Each hand was placed on a handle, with the index fingers positioned in the circular grooves of rotatable dials (Supplemental Figure 1.C). While maintaining contact with the handles, only the index fingers could move.

Each trial began with a blue target line on a black background that remained visible until the end of each trial. Participants were instructed to start moving as soon as the yellow dot disappeared, rotating the dials to control vertical (left dial) and horizontal (right dial) cursor movements, while a red line visualized the real-time trajectory of the cursor (Trajectory feedback; Supplemental Figure 1.A). The movement phase ended when the white dot reached the opposite end of the blue target line.

# Supplement 2. MRSinMRS checklist

**MRSinMRS checklist – minimum reporting standards (Lin et al., 2021)**

| 1. **Hardware** |  |
| --- | --- |
| 1. Field strength | 3 T |
| 1. Manufacturer | Philips |
| 1. Model | Achieva dstream |
| 1. RF coils: nuclei (transmit/receive), number of channels, type, body part | 32-channel receiver head coil (Philips, Best, The Netherlands) |
| 1. Additional hardware | N/A |
| 1. **Acquisition** |  |
| 1. Pulse sequence | MEGA Press |
| 1. Volume of interest (VOI) locations | Right Cerebellar hemisphere |
| 1. Nominal VOI size AP x LR x HF(cm^3^, mm^3^) | 30 x 25 x 25 mm^3^ |
| 1. Repetition time (T_R_), echo time (T_E_) (ms,s) | GABA: T_R_ = 2000 ms ; T_E_ = 68 ms  GSH: T_R_ = 2000 ms ; T_E_ = 120 |
| 1. Total number of excitations of acquisitions per spectrum | 320 averages |
| 1. Additional sequence parameters (spectral width in Hz, number of spectral points, frequency offsets) | 2000 Hz; 1024 points; N/A |
| 1. Water suppression method | Multiply Optimized Insensitive Suppression Train (MOIST; bandwidth 140 Hz) |
| 1. Shimming method, reference peak, and thresholds for “acceptance of shim” chosen | Automated “pencil-beam” (PB) shimming procedure (Philips) |
| 1. Triggering or motion correction method | N/A |
| 1. **Data analysis methods and outputs** |  |
| 1. Analysis software | Gannet 3.4.0-dev |
| 1. Processing steps deviating from the quoted reference or product | GSH: frequency alignment; data-driven approach described in Supplement 3 |
| 1. Output measure | TissCorWater scaled (Gasparovic) in institutional units (i.u.) |
| 1. Quantification references and assumptions, fitting model assumptions | 3-Gaussian using a nonlinear baseline |
| 1. **Data quality** |  |
| 1. Reported variables (SNR, FWHM) | FWHM_GABA: YA, 18.79±2.00 / OA, 19.15±2.21; FWHM_Glx: YA, 14.44±1.44 / OA, 14.63±2.33, FWHM Cr: YA, 8.66±0.89 / OA, 9.44±1. 85; FWHM_GSH: YA, 15.06±3.39 / OA, 14.58±4.38; SNR_GABA: YA, 12.69±2.04 / OA, 11.24±1.92; SNR_Glx: YA, 15.58±3.24 / OA, 13.87±2.71; SNR_GSH: YA 6.86±1.41 / OA: 7.38±1.60; See Table 2 of the manuscript |
| 1. Add data exclusion criteria | Visual inspection: out-of-voxel echoes, lipid contamination;  If no clear GABA+ or GSH signal could be detected or if Gannet modeling of the data failed;  Scaled estimates for GABA+ and GSH that exceeded five times the median absolute deviation (MAD) from the median, ensuring the detection of poor fits not captured by other criteria (Rousseeuw and Hubert, 2011; Craven et al., 2022). |
| 1. Quality measures of postprocessing model fitting (e.g., CRLB, goodness of fit, SD of residual) | Fit Error_GABA: YA, 6.30±1.27 / OA, 6.86±2.01; Fit Error_Glx: YA, 4.80±2.29 / OA, 5.69±2.91; Fit Error_GSH: YA, 13.15±3.57 / OA, 15.19±5.07 |
| 1. Sample spectrum | See Figure 2 of the manuscript |

# Supplement 3. GSH analysis

A data driven approach was used for the GSH analysis. Specifically, data quality was assessed through visual inspection of the NAA-corrected spectra, as well as the fit error, signal- to- noise ratio (SNR), and full- width- half- maximum (FWHM) of GSH and FWHM of NAA. If poor quality was due to misalignment, spectral correction was applied using robust spectral correction (RobustSpecReg; one older adult) or, alternatively, no alignment correction was applied (six older and five younger adults). GSH data were excluded for three older and two younger participants due to insufficient quality.

The mean difference spectra for both age groups, along with standard deviation (SD) and confidence intervals (CI), are presented for each alignment option apart (see Supplemental Figure 2).


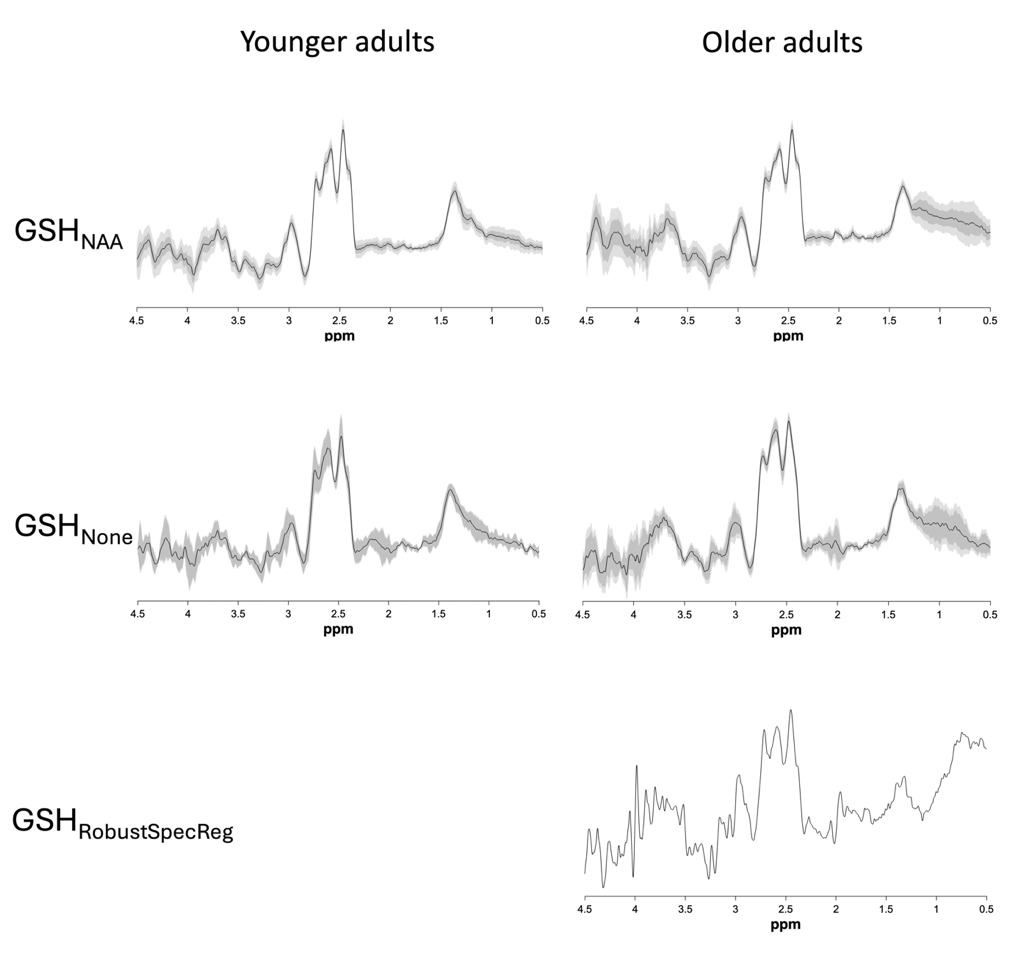


***Supplemental Figure 2.*** *Spectral overlay. Difference spectra (mean±SD and 90% CI) per age group for GSH. Plots are being displayed for each alignment method apart.*

***Abbreviations:*** *GSH = glutathione; Ppm = parts per million*

To look at the potential influence of alignment (NAA, RobustSpecReg and none) as well as sex, a multiple regression analysis was used. Specifically, GSH was added as the dependent variable, while age group (YA and OA), sex (female and male) and alignment (NAA, RobustSpecReg and none) were added as independent variables.

To comply with model assumptions, a square root transformation of the dependent variable was conducted. The model explained a significant proportion of the variance in GSH of R^2^=0.13, F(4,40) = 1.52, *p* = 0.2156. None of the predictors contributed significantly to the model (all p < 0.05) (Supplementary Table 1).

***Supplementary Table 1.*** *Multiple regression model GSH*

| **Variable** | **DF** | **Sum of squares** | **F ratio** | **Prob > F** |
| --- | --- | --- | --- | --- |
| Age group | 1 | 0.1664 | 1.49 | 0.2286 |
| Sex | 1 | 0.0036 | 0.03 | 0.8578 |
| Alignment | 2 | 0.5334 | 2.40 | 0.1040 |

While some studies have shown sex-related differences in GSH levels in both younger (Mandal et al., 2012; Lally et al., 2016) and older adults (Michels et al., 2024), we did not find any differences between men and women in cerebellar GSH levels. This is in line with e.g., Busler et al. (2023). This inconsistency between different studies may be due to the fact that different brain regions were investigated–for instance, possible sex-related differences in GSH levels have never been investigated in the cerebellum–as well as the different methodologies used.

# Supplement 4: Exploratory Glx/GABA+ ratio (E/I Balance)

# To complement the primary analyses, we calculated the Glx/GABA+ ratio as a proxy for excitatory/inhibitory balance at each MRS acquisition time point. While this ratio was not used in the main statistical models due to its dependency on both individual components, we report it here to provide additional insight into potential group differences in neurochemical balance.

***Supplementary Table 2.*** *Group comparison normalized modulation*

|  | Younger adults  (median±IQR)  (mean±SD) ∇ | Older adults  (median±IQR)  (mean±SD) ∇ | Group comparison  p-value of WRST  p-value of t-test ∇ |
| --- | --- | --- | --- |
| Glx/GABA+_pre | 3.14±0.43∇ | 3.10±0.41∇ | 0.7289∇ |
| Glx/GABA+_BTT | 3.38±0.60 | 3.35±0.56∇ | 0.8462 |
| Glx/GABA+_post | 3.14±0.67∇ | 3.15±0.37∇ | 0.9948∇ |

***Abbreviations:*** *BTT = bimanual tracking taks; GABA = gaba aminobutyric acid; Glx = combined glutamate and glutamine level; WRST = Wilcoxon rank-sum test*

# Supplement 5: Alternative correction strategies GABA

Alternative to the Gasparovic correction (Gasparovic et al., 2006) for partial volume effects, we introduce two additional correction strategies to ensure that the absence of age-related differences in GABA reported in the manuscript is not an artifact of the chosen correction method.

## GABAWater_CSF corrected

Similar to the analysis described in the manuscript changes in GABA levels during task performance and potential group differences in modulation were assessed using a linear mixed model (LMM). GABA was added as the dependent variable, and age group (YA and OA), timepoint (pre, BTT, and post), and age group*timepoint as fixed effects. Participant was included as a random effect. Here, we used the CSF-only correction and included the GM/WM ratio as a covariate of no interest. The GABA_post_ data of one young adult was excluded from the analysis because its residual represented a visual outlier on the Q-Q plot.

There were no significant effects of neither timepoint (pre, BTT, and post levels), age group, or their interaction, nor GM/WM ratio on GABA levels (all *p* > 0.05).

## GABAWater_uncorrected

Next, an LMM with uncorrected GABA levels as dependent variable and age group (YA and OA), timepoint (pre, BTT, and post), and age group*timepoint included as fixed effects was created. Participant was included as a random effect. The GABA_post_ data of one young adult was excluded from the analysis because its residual represented a visual outlier on the Q-Q plot.

Uncorrected GABA levels were not significantly influenced by timepoint (pre, BTT, and post levels), age group, or their interaction (all p > 0.05)

# Supplement 6: Multiple regression models association between motor performance and short-term motor learning and task-related GABA modulation, Glx modulation, and baseline neurometabolite levels

## Baseline neurometabolite levels

### Performance

The associations between motor performance, and baseline neurometabolite levels (GABA, Glx and GSH) were explored using multiple regression models. Each model included fixed effects for age group, GABA_pre_, Glx_pre_, GSH, age group*GABA_pre_, age group*Glx_pre_, age group*GSH, GABA_pre_*Glx_pre_, GABA_pre_*GSH and Glx_pre_*GSH. Sex was added as a covariate of no interest.

***Supplementary Table 3.*** *Parameter estimates full model performance by baseline neurometabolite levels*

| **Term** | **Estimate** | **Std Error** | **t Ratio** | **Prob>\|t\|** |
| --- | --- | --- | --- | --- |
| Intercept | 137.74 | 155.74 | 0.88 | 0.3825 |
| Sex[F] | -2.76 | 1.32 | -2.10 | **0.0435*** |
| Group[OA] | -3.87 | 18.09 | -0.21 | 0.8319 |
| GABA_pre_ | -39.31 | 34.45 | -1.14 | 0.2617 |
| Glx_pre_ | 2.84 | 10.00 | 0.28 | 0.7779 |
| GSH | -16.05 | 18.86 | -0.85 | 0.4003 |
| Group[OA]*GABA_pre_ | 0.63 | 2.65 | 0.24 | 0.8149 |
| Group[OA]*Glx_pre_ | -0.66 | 1.05 | -0.62 | 0.5368 |
| Group[OA]*GSH | -0.19 | 1.046 | -0.19 | 0.8537 |
| GABA_pre_*Glx_pre_ | 0.51 | 2.14 | 0.24 | 0.8119 |
| GABA_pre_*GSH | 7.31 | 3.92 | 1.86 | 0.0707 |
| Glx_pre_*GSH | -1.33 | 0.68 | -1.96 | 0.0578 |

***Abbreviations:*** *F = female; GABA = gaba aminobutyric acid; Glx = combined glutamate and glutamine level; GSH = glutathione; OA = older adults*

***Supplementary Table 4.*** *Effect tests full model performance by baseline neurometabolite levels*

| **Source** | **Nparm** | **DF** | **Sum of Squares** | **F Ratio** | **Prob > F** |
| --- | --- | --- | --- | --- | --- |
| Sex | 1 | 1 | 296.74 | 4.39 | **0.0435*** |
| Group | 1 | 1 | 3.09 | 0.05 | 0.8319 |
| GABA_pre_ | 1 | 1 | 87.98 | 1.30 | 0.2617 |
| Glx_pre_ | 1 | 1 | 5.46 | 0.08 | 0.7779 |
| GSH | 1 | 1 | 49.00 | 0.73 | 0.4003 |
| Group*GABA_pre_ | 1 | 1 | 3.76 | 0.06 | 0.8149 |
| Group*Glx_pre_ | 1 | 1 | 26.30 | 0.39 | 0.5368 |
| Group*GSH | 1 | 1 | 2.33 | 0.03 | 0.8537 |
| GABA_pre_*Glx_pre_ | 1 | 1 | 3.89 | 0.06 | 0.8119 |
| GABA_pre_*GSH | 1 | 1 | 234.91 | 3.48 | 0.0707 |
| Glx_pre_*GSH | 1 | 1 | 260.13 | 3.85 | 0.0578 |

***Abbreviations:*** *GABA = gaba aminobutyric acid; Glx = combined glutamate and glutamine level; GSH = glutathione*

***Supplementary Table 5.*** *Parameter estimates final model performance by baseline neurometabolite levels*

| **Term** | **Estimate** | **Std Error** | **T Ratio** | **Prob>\|t\|** |
| --- | --- | --- | --- | --- |
| Intercept | 26.64 | 1.28 | 20.78 | **<0.0001*** |
| Sex[F] | -3.53 | 1.28 | -2.75 | **0.0085*** |
| Group[OA] | -10.14 | 1.28 | -7.93 | **<0.0001*** |

***Abbreviations:*** *F = female; OA = older adults*

***Supplementary Table 6.*** *Effect tests final model performance by baseline neurometabolite levels*

| **Source** | **Nparm** | **DF** | **Sum of Squares** | **F Ratio** | **Prob > F** |
| --- | --- | --- | --- | --- | --- |
| Sex | 1 | 1 | 592.10 | 7.57 | **0.0085*** |
| Group | 1 | 1 | 4918.92 | 62.89 | **<0.0001*** |

### Short-term motor learning

The associations between short-term motor learning, and baseline neurometabolite levels (GABA, Glx and GSH) were explored using multiple regression models. Each model included fixed effects for age group, GABA_pre_, Glx_pre_, GSH, age group*GABA_pre_, age group*Glx_pre_, age group*GSH, GABA_pre_*Glx_pre_, GABA_pre_*GSH and Glx_pre_*GSH. Sex was added as a covariate of no interest.

***Supplementary Table 7.*** *Parameter estimates full model short-term motor learning by baseline neurometabolite levels*

| **Term** | **Estimate** | **Std Error** | **t Ratio** | **Prob>\|t\|** |
| --- | --- | --- | --- | --- |
| Intercept | 4.05 | 3.44 | 1.18 | 0.2461 |
| Sex[F] | -0.02 | 0.03 | -0.66 | 0.5108 |
| Group[OA] | -0.40 | 0.41 | -0.96 | 0.3419 |
| GABA_pre_ | -1.02 | 0.76 | -1.34 | 0.1888 |
| Glx_pre_ | -0.13 | 0.22 | -0.59 | 0.5592 |
| GSH | -0.23 | 0.43 | -0.52 | 0.6048 |
| Group[OA]*GABA_pre_ | 0.00 | 0.06 | 0.06 | 0.9524 |
| Group[OA]*Glx_pre_ | 0.02 | 0.02 | 1.07 | 0.2932 |
| Group[OA]*GSH | -0.01 | 0.02 | -0.45 | 0.6522 |
| GABA_pre_*Glx_pre_ | 0.04 | 0.05 | 0.86 | 0.3949 |
| GABA_pre_*GSH | 0.10 | 0.09 | 1.08 | 0.2862 |
| Glx_pre_*GSH | -0.02 | 0.02 | -0.99 | 0.3294 |

***Abbreviations****: F = female; GABA = gaba aminobutyric acid; Glx = combined glutamate and glutamine level; GSH = glutathione; OA = older adults*

***Supplementary Table 8.*** *Effect tests full model short-term motor learning by baseline neurometabolite levels*

| **Source** | **Nparm** | **DF** | **Sum of Squares** | **F Ratio** | **Prob > F** |
| --- | --- | --- | --- | --- | --- |
| Sex | 1 | 1 | 0.02 | 0.44 | 0.5108 |
| Group | 1 | 1 | 0.03 | 0.93 | 0.3419 |
| GABA_pre_ | 1 | 1 | 0.06 | 1.79 | 0.1888 |
| Glx_pre_ | 1 | 1 | 0.01 | 0.35 | 0.5592 |
| GSH | 1 | 1 | 0.01 | 0.27 | 0.6048 |
| Group*GABA_pre_ | 1 | 1 | 0.00 | 0.00 | 0.9524 |
| Group*Glx_pre_ | 1 | 1 | 0.04 | 1.14 | 0.2932 |
| Group*GSH | 1 | 1 | 0.01 | 0.21 | 0.6522 |
| GABA_pre_*Glx_pre_ | 1 | 1 | 0.03 | 0.74 | 0.3949 |
| GABA_pre_*GSH | 1 | 1 | 0.04 | 1.17 | 0.2862 |
| Glx_pre_*GSH | 1 | 1 | 0.04 | 0.98 | 0.3294 |

***Abbreviations:*** *GABA = Gaba aminobutyric acid; Glx = combined glutamate and glutamine level; GSH = glutathione*

***Supplementary Table 9.*** *Parameter estimates final model short-term motor learning by baseline neurometabolite levels*

| **Term** | **Estimate** | **Std Error** | **t Ratio** | **Prob>\|t\|** |
| --- | --- | --- | --- | --- |
| Intercept | 0.18 | 0.02 | 7.14 | **<0.0001*** |
| Sex[F] | -0.01 | 0.02 | -0.52 | 0.6054 |
| Group[OA] | -0.06 | 0.02 | -2.26 | **0.0287*** |

***Abbreviations:*** *F = female; OA = older adults*

***Supplementary Table 10.*** *Effect tests final model short-term motor learning by baseline neurometabolite levels*

| **Source** | **Nparm** | **DF** | **Sum of Squares** | **F Ratio** | **Prob > F** |
| --- | --- | --- | --- | --- | --- |
| Sex | 1 | 1 | 0.01 | 0.27 | 0.6054 |
| Group | 1 | 1 | 0.16 | 5.09 | **0.0287*** |

## GABA/Glx modulation

### Performance

Similarly, the relationship between motor performance, and task-related GABA/Glx modulation were examined using multiple regression models. These models included fixed effects for age group, modulation GABA, modulation Glx, group*modulation GABA, age group*modulation Glx, and modulation GABA*modulation Glx. Sex was added again as a covariate of no interest. Separate models were constructed for two behavioral outcomes: performance and short-term learning.

***Supplementary Table 11.*** *Parameter estimates full model performance by GABA/Glx modulation*

| **Term** | **Estimate** | **Std Error** | **t Ratio** | **Prob>\|t\|** |
| --- | --- | --- | --- | --- |
| Intercept | 25.18 | 1.67 | 15.09 | **<0.0001*** |
| Sex[F] | -2.32 | 1.50 | -1.55 | 0.1289 |
| Group[OA] | -8.23 | 1.77 | -4.65 | **<0.0001*** |
| Modulation GABA | -0.91 | 3.17 | -0.29 | 0.7755 |
| Modulation Glx | 1.35 | 1.19 | 1.13 | 0.2635 |
| Group[OA]*Modulation GABA | 1.05 | 3.22 | 0.33 | 0.746 |
| Group[OA]*Modulation Glx | -1.00 | 1.17 | -0.86 | 0.3969 |
| Modulation GABA*Modulation Glx | -1.38 | 1.60 | -0.86 | 0.3929 |

***Abbreviations:*** *F = female; GABA = gaba aminobutyric acid; Glx = combined glutamate and glutamine level; GSH = glutathione; OA = older adults*

***Supplementary Table 12.*** *Effect tests full model performance by GABA/Glx modulation*

| **Source** | **Nparm** | **DF** | **Sum of Squares** | **F Ratio** | **Prob > F** |
| --- | --- | --- | --- | --- | --- |
| Sex | 1 | 1 | 259.94 | 2.40 | 0.1289 |
| Group | 1 | 1 | 2347.55 | 21.66 | **<0.0001*** |
| Modulation GABA | 1 | 1 | 8.93 | 0.08 | 0.7755 |
| Modulation Glx | 1 | 1 | 139.21 | 1.28 | 0.2635 |
| Group*Modulation GABA | 1 | 1 | 11.52 | 0.10 | 0.746 |
| Group*Modulation Glx | 1 | 1 | 79.39 | 0.73 | 0.3969 |
| Modulation GABA*Modulation Glx | 1 | 1 | 80.74 | 0.75 | 0.3929 |

***Abbreviations:*** *GABA = gaba aminobutyric acid; Glx = combined glutamate and glutamine level; GSH = glutathione*

***Supplementary Table 13.*** *Parameter estimates final model performance by GABA/Glx modulation*

| **Term** | **Estimate** | **Std Error** | **t Ratio** | **Prob>\|t\|** |
| --- | --- | --- | --- | --- |
| Intercept | 25.46 | 1.45 | 17.57 | **<0.0001*** |
| Sex[F] | -2.35 | 1.45 | -1.62 | 0.1119 |
| Group[OA] | -9.01 | 1.45 | -6.22 | **<0.0001*** |

***Supplementary Table 14.*** *Effect tests final model performance by GABA/Glx modulation*

| **Source** | **Nparm** | **DF** | **Sum of Squares** | **F Ratio** | **Prob > F** |
| --- | --- | --- | --- | --- | --- |
| Sex | 1 | 1 | 275.15 | 2.63 | 0.1119 |
| Group | 1 | 1 | 4058.32 | 38.72 | **<0.0001*** |

### Short-term motor learning

The relationship between short-term learning, and task-related GABA/Glx modulation were examined using multiple regression models. These models included fixed effects for age group, modulation GABA, modulation Glx, group*modulation GABA, age group*modulation Glx, and modulation GABA*modulation Glx. Sex was added again as a covariate of no interest. Separate models were constructed for two behavioral outcomes: performance and short-term learning.

***Supplementary Table 15.*** *Parameter estimates full model short-term motor learning by GABA/Glx modulation*

| **Term** | **Estimate** | **Std Error** | **t Ratio** | **Prob>\|t\|** |
| --- | --- | --- | --- | --- |
| Intercept | 0.18 | 0.03 | 6.21 | **<0.0001*** |
| Sex[F] | -0.01 | 0.03 | -0.36 | 0.7221 |
| Group[OA] | -0.05 | 0.03 | -1.61 | 0.1150 |
| Modulation GABA | -0.01 | 0.06 | -0.12 | 0.9039 |
| Modulation Glx | -0.01 | 0.02 | -0.28 | 0.7777 |
| Group[OA]*Modulation GABA | 0.03 | 0.06 | 0.59 | 0.5570 |
| Group[OA]*Modulation Glx | 0.01 | 0.02 | 0.62 | 0.5386 |
| Modulation GABA*Modulation Glx | -0.02 | 0.03 | -0.81 | 0.4250 |

***Supplementary Table 16.*** *Effect tests full model short-term motor learning by GABA/Glx modulation*

| **Source** | **Nparm** | **DF** | **Sum of Squares** | **F Ratio** | **Prob > F** |
| --- | --- | --- | --- | --- | --- |
| Sex | 1 | 1 | 0.00 | 0.13 | 0.7221 |
| Group | 1 | 1 | 0.09 | 2.59 | 0.1150 |
| Modulation GABA | 1 | 1 | 0.00 | 0.01 | 0.9039 |
| Modulation Glx | 1 | 1 | 0.00 | 0.08 | 0.7777 |
| Group*Modulation GABA | 1 | 1 | 0.01 | 0.35 | 0.5570 |
| Group*Modulation Glx | 1 | 1 | 0.01 | 0.38 | 0.5386 |
| Modulation GABA*Modulation Glx | 1 | 1 | 0.02 | 0.65 | 0.4250 |

***Supplementary Table 17.*** *Parameter estimates final model short-term motor learning by GABA/Glx modulation*

| **Term** | **Estimate** | **Std Error** | **t Ratio** | **Prob>\|t\|** |
| --- | --- | --- | --- | --- |
| Intercept | 0.18 | 0.02 | 7.14 | **<0.0001*** |
| Sex[F] | -0.01 | 0.02 | -0.52 | 0.6054 |
| Group[OA] | -0.06 | 0.02 | -2.26 | **0.0287*** |

***Abbreviations:*** *F = female; OA = older adults*

***Supplementary Table 18.*** *Effect tests final model short-term motor learning by GABA/Glx modulation*

| **Source** | **Nparm** | **DF** | **Sum of Squares** | **F Ratio** | **Prob > F** |
| --- | --- | --- | --- | --- | --- |
| Sex | 1 | 1 | 0.01 | 0.27 | 0.6054 |
| Group | 1 | 1 | 0.16 | 5.09 | **0.0287*** |

# References

Busler, J.N., Slate, S.R., Liao, H., Lyndon, S., Taylor, J., Lin, A.P., et al. (2023). Sex hormones as correlates of oxidative stress in the adult brain. *Psychiatry Res Neuroimaging* 334**,** 111681. doi: 10.1016/j.pscychresns.2023.111681.

Craven, A.R., Bhattacharyya, P.K., Clarke, W.T., Dydak, U., Edden, R.A.E., Ersland, L., et al. (2022). Comparison of seven modelling algorithms for γ-aminobutyric acid-edited proton magnetic resonance spectroscopy. *NMR Biomed* 35(7)**,** e4702. doi: 10.1002/nbm.4702.

Gasparovic, C., Song, T., Devier, D., Bockholt, H.J., Caprihan, A., Mullins, P.G., et al. (2006). Use of tissue water as a concentration reference for proton spectroscopic imaging. *Magnetic Resonance in Medicine* 55(6)**,** 1219-1226. doi: <https://doi.org/10.1002/mrm.20901>.

Lally, N., An, L., Banerjee, D., Niciu, M.J., Luckenbaugh, D.A., Richards, E.M., et al. (2016). Reliability of 7T (1) H-MRS measured human prefrontal cortex glutamate, glutamine, and glutathione signals using an adapted echo time optimized PRESS sequence: A between- and within-sessions investigation. *J Magn Reson Imaging* 43(1)**,** 88-98. doi: 10.1002/jmri.24970.

Lin, A., Andronesi, O., Bogner, W., Choi, I.Y., Coello, E., Cudalbu, C., et al. (2021). Minimum Reporting Standards for in vivo Magnetic Resonance Spectroscopy (MRSinMRS): Experts' consensus recommendations. *NMR Biomed* 34(5)**,** e4484. doi: 10.1002/nbm.4484.

Mandal, P.K., Tripathi, M., and Sugunan, S. (2012). Brain oxidative stress: Detection and mapping of anti-oxidant marker ‘Glutathione’ in different brain regions of healthy male/female, MCI and Alzheimer patients using non-invasive magnetic resonance spectroscopy. *Biochemical and Biophysical Research Communications* 417(1)**,** 43-48. doi: <https://doi.org/10.1016/j.bbrc.2011.11.047>.

Michels, L., O’Gorman-Tuura, R., Bachmann, D., Müller, S., Studer, S., Saake, A., et al. (2024). The links among age, sex, and glutathione: A cross-sectional magnetic resonance spectroscopy study. *Neurobiology of Aging* 144**,** 19-29. doi: <https://doi.org/10.1016/j.neurobiolaging.2024.08.010>.

Rousseeuw, P.J., and Hubert, M. (2011). Robust statistics for outlier detection. *WIREs Data Mining and Knowledge Discovery* 1(1)**,** 73-79. doi: <https://doi.org/10.1002/widm.2>.
